# Emergent Network Topology within the Respiratory Rhythm-Generating Kernel Evolved *In Silico*

**DOI:** 10.1371/journal.pone.0154049

**Published:** 2016-05-06

**Authors:** Amit Lal, Yoshitaka Oku, Hiroshi Someya, Fumikazu Miwakeichi, Yoshiyasu Tamura

**Affiliations:** 1 Department of Biomedical Engineering, Peking University, Beijing, 100871 P. R. China; 2 Department of Physiology, Hyogo College of Medicine, Nishinomiya, 663-8501 Japan; 3 School of Information Science and Technology, Tokai University, Hiratsuka, 259-1292 Japan; 4 Department of Statistical Modeling, The Institute of Statistical Mathematics, Tachikawa, 190-8562 Japan; 5 Department of Statistical Science, School of Multidisciplinary Sciences, The Graduate University for Advanced Studies, Tachikawa, 190-8562, Japan; McGill University, CANADA

## Abstract

We hypothesize that the network topology within the pre-Bötzinger Complex (preBötC), the mammalian respiratory rhythm generating kernel, is not random, but is optimized in the course of ontogeny/phylogeny so that the network produces respiratory rhythm efficiently and robustly. In the present study, we attempted to identify topology of synaptic connections among constituent neurons of the preBötC based on this hypothesis. To do this, we first developed an effective evolutionary algorithm for optimizing network topology of a neuronal network to exhibit a ‘desired characteristic’. Using this evolutionary algorithm, we iteratively evolved an *in silico* preBötC ‘model’ network with initial random connectivity to a network exhibiting optimized synchronous population bursts. The evolved ‘idealized’ network was then analyzed to gain insight into: (1) optimal network connectivity among different kinds of neurons—excitatory as well as inhibitory pacemakers, non-pacemakers and tonic neurons—within the preBötC, and (2) possible functional roles of inhibitory neurons within the preBötC in rhythm generation. Obtained results indicate that (1) synaptic distribution within excitatory subnetwork of the evolved model network illustrates skewed/heavy-tailed degree distribution, and (2) inhibitory subnetwork influences excitatory subnetwork primarily through non-tonic pacemaker inhibitory neurons. Further, since small-world (SW) network is generally associated with network synchronization phenomena and is suggested as a possible network structure within the preBötC, we compared the performance of SW network with that of the evolved model network. Results show that evolved network is better than SW network at exhibiting synchronous bursts.

## 1 Introduction

It is long identified that the kernel of the mammalian respiratory rhythm generation is a neuronal organization called the pre-Bötzinger Complex (preBötC) located in the ventrolateral region of the medulla [1]. Although respiratory rhythmogenesis *in vivo* involves the broader respiratory network consisting of pontine respiratory neurons and Bötzinger complex [2], the precise mechanism how the microcircuit within the preBötC generates rhythmic behavior even in reduced states of network is still unknown. To facilitate understanding of the mechanism of rhythm generation by the preBötC, behaviors of constituent neurons have been primarily investigated at two distinct levels: 1) network behavior that has classified neurons as inspiratory, pre-inspiratory, post-inspiratory, and expiratory (e.g. [3]), and 2) intrinsic cellular behavior (observed after pharmacologically blocking synaptic transmission among network neurons) that has classified neurons as pacemakers, non-pacemaker, and tonic (e.g. [4]). It is reported that pacemaker neurons within the preBötC are both excitatory and inhibitory in nature [5]. Respiratory rhythm persists even when inhibitory synaptic transmission is pharmacologically blocked in breathing slices of neonatal rats [6]. Nevertheless, it is demonstrated that inhibitory neurons are functionally integrated within the rhythm-generating network [5, 7]. The roles of these inhibitory preBötC neurons in rhythmogenesis remain to be clarified.

With these recent findings, of primary importance to respiratory neurophysiologist is to be able to explain the network behavior of constituent neurons of the preBötC as an emergent property of their intrinsic behavior; thereby establishing the mechanism of rhythm generation in the preBötC. To this end, simulation studies have helped to explain various specific aspects of this emergent phenomena [4, 8–13]. However, the aspect of preBötC neuronal group which has remained unexplored in this regard is the topological structure of synaptic connections among its constituent neurons. Since population activity of a neuronal group is determined by both its constituent neurons and topology of network connectivity, even if characteristics of constituent neurons of the preBötC are known, in absence of knowledge of synaptic connection pattern among them, the precise determination of rhythm generation at population level would be incomplete. Indeed, recent findings suggest that the neuronal network topology plays a vital role in determining overall network behavior [14, 15].

Computational neurophysiologists have generally modeled preBötC neuronal groups assuming all-to-all connectivity among the constituent neurons [4, 8, 9, 13]. However, assuming all-to-all connectivity among the constituent neurons within a neuronal group is unrealistic and lacks even the essential qualitative features of real network. We have highlighted some of the limitations of all-to-all connectivity in [16] and illustrated a way of overcoming it. This might be one of the reasons why the possibility of quantal slowing mechanism due to intermittent failure of preBötC neurons to synchronize and exhibit bursting in unison, was missed by earlier studies [17]. Thus, identification of the pattern of synaptic connectivity among the different types of neurons within the preBötC holds the key to achieve the understanding of the functional roles played by these constituent neurons in the rhythm generation.

Fraction of bursting neurons within preBötC neuronal group is reported to be 10–15% [4]. Further, a recent simulation study [3] has suggested that only sparsely connected neuronal network (near 1%) is able to reliably reproduce experimentally observed burst onset variability of preBötC neurons quantitatively. Although direct experimental investigation of network topology is technically difficult, attempts have been made to study network topology through imaging [18, 19]. Based on cluster analysis of imaging results, it was suggested that network topology within the preBötC may well be a small-world [20] or scale-free [21], emphasizing the need of further study in this direction.

We hypothesize that the network topology within the preBötC is not random, but is optimized in the course of ontogeny/phylogeny so that the network produces the respiratory rhythm efficiently and robustly. In the present study, we first develop an effective evolutionary algorithm for evolving the network topology of a neuronal network to exhibit a desired characteristic. Using this evolutionary algorithm as a tool, we iteratively simulate the behavior of a preBötC ‘model’ network while mutating its network topology so as to help it evolve to a network exhibiting optimized synchronous population bursts, with the specific aim of gaining insight into: 1) the optimal topology of neuronal connectivity among different kinds of neurons within the preBötC neuronal group, and 2) possible functional roles of inhibitory neurons within the preBötC in rhythm generation. Since small-world (SW) network is one of the most common network studied in context of network synchronization phenomena [20], and is often suggested as a possible network structure within the preBötC [18], we further evaluate the performance of SW network in terms of synchronicity, and compare it with that of the optimally evolved network.

## 2 Method

### 2.1 Concept

**Fig 1:**
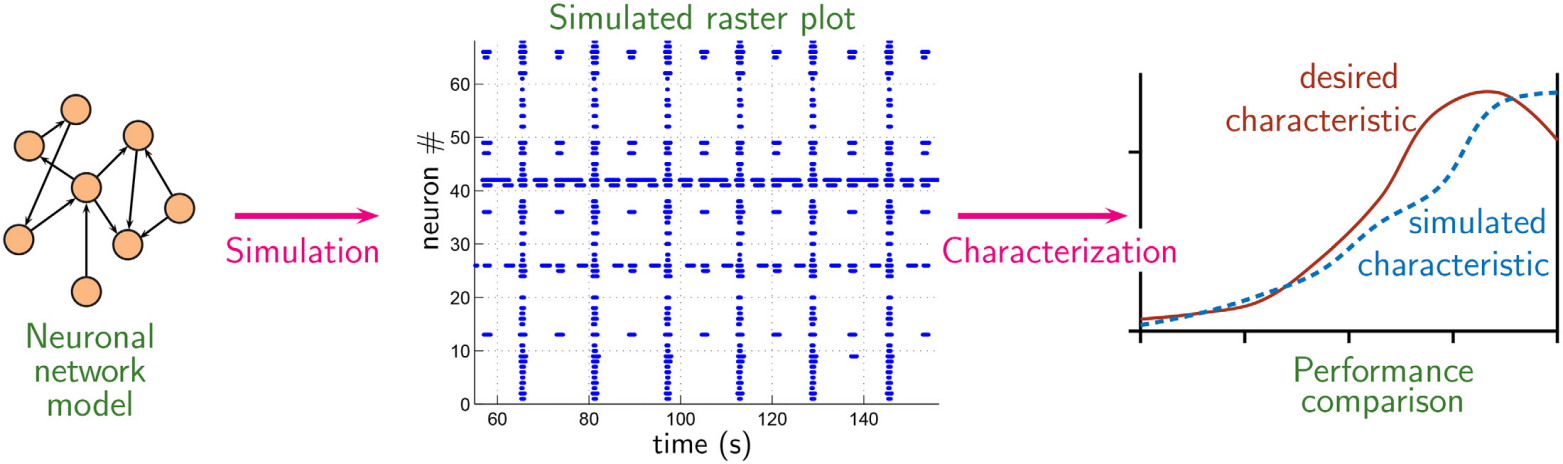
Method concept. For a mathematical model neuronal network with fixed intrinsic parameters of constituting neurons, its group behavior is a function of network topology alone. Different connection topology of synapses among neurons of network produce different group behavior. Group behavior may be visualized in terms of a raster plot from which essential feature may be subsequently extracted in the form of a ‘characteristic curve’. By employing an evolutionary algorithm, network topology is iteratively evolved so that its characteristic curve matches a ‘desired characteristic’.

Considering a neuronal network as a dynamical system, it is clear that its behavior is critically determined by intrinsic parameters of its constituting neurons and the connection topology of synapses among them. Once neuronal parameter values are fixed, behaviour of neuronal network becomes solely a function of connection topology of synapses among the neurons. Different connection topology of synapses among network neurons produce different network behavior [14]. As is depicted in Fig 1, the neuronal activity of the network can be simulated and its behavior be characterized by a ‘characteristic curve’ (see section 2.1.1 below). This characteristic curve differs for different connection topology of synapses among neurons of the network. By employing an evolutionary algorithm, network topology is iteratively evolved so that its characteristic curve matches a ‘desired characteristic’. The connection topology of the evolved network is subsequently examined and its important features are identified.

It is remarked that the ‘desired characteristic’ curve depicted in Fig 1 may be an experimentally observed characteristic or an abstract idealization of it, depending on the intended subject of investigation.

#### 2.1.1 Definition of ‘Characteristic curve’ and ‘desired characteristic’

In the present work, we aim to identify connection topology which is better suited for synchronizing the bursting activities of constituting neurons, and thus enabling the network to exhibit robust population bursts.

**Fig 2:**
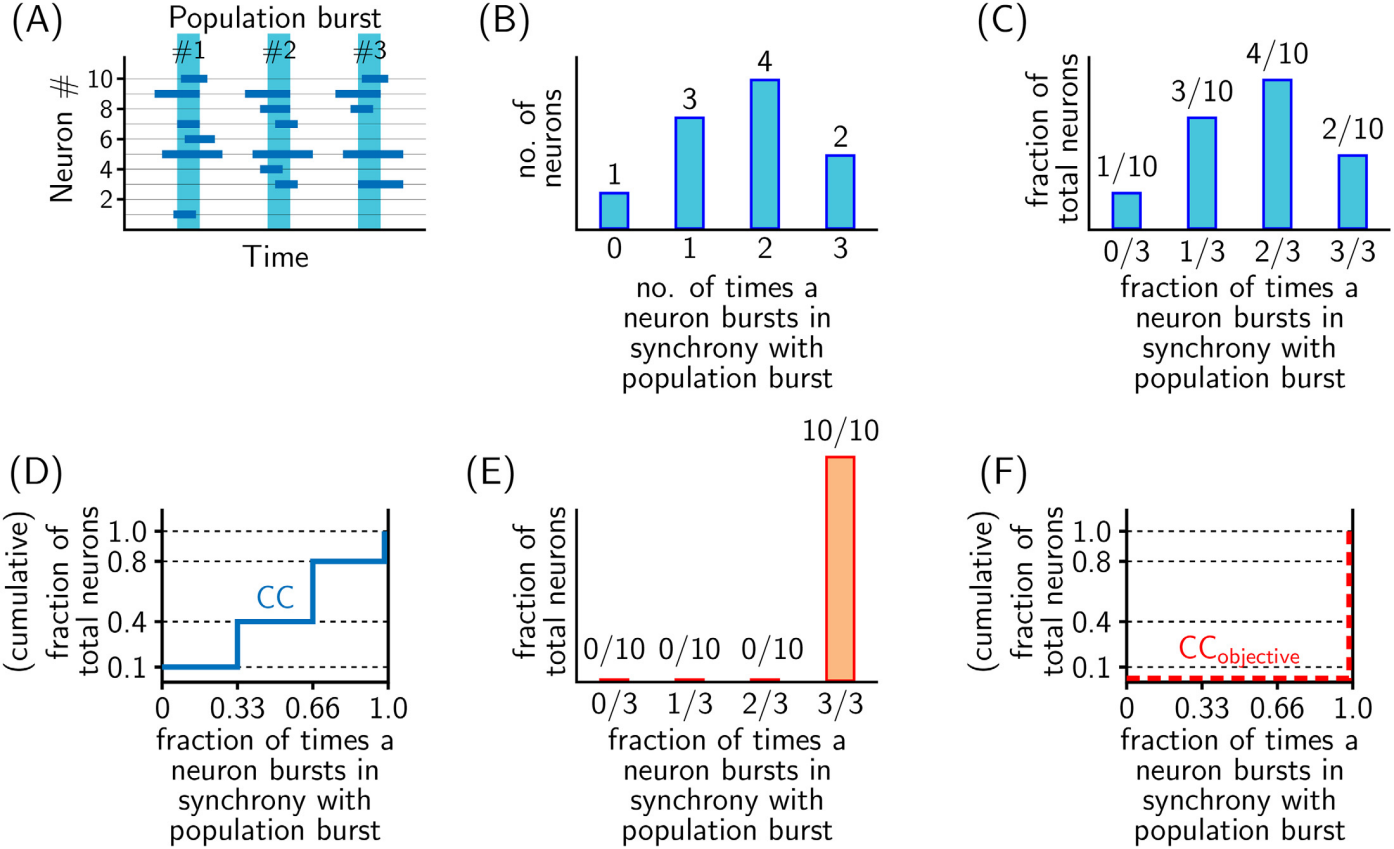
Procedure for computing cumulative curves (CC) to measure network synchronicity. Illustrated for a hypothetical case (A), when: total number of network neurons = 10, total observed/recorded population burst = 3, total number of neurons that burst in synchrony with population burst once (out of 3 observed population burst), twice, thrice and never are equal to 3 (neuron #1, 4, and 6), 4 (neuron #3, 7, 8 and 10), 2 (neuron #5 and 9) and 1 (neuron #2), respectively. (B) Frequency distribution depicting number of times various neurons participate in population burst. (C) Normalized representation of frequency distribution in (B). (D) Cumulative curve obtained from frequency distribution in (C). (E) Normalized frequency distribution for a completely synchronized network; all neurons participating in every population burst. (F) Cumulative curve (CC_objective_) obtained from (E).

As is depicted in Fig 1, network behavior may be visualized in terms of *simulated* raster plot (which is time history of bursting activity all constituting neurons of the network). In the present work, we characterized the ‘simulated raster plot’ in terms of cumulative curves (CC) as illustrated in Fig 2. Cumulative curves (CC) in Fig 2 characterizes synchronicity of bursting activities of constituent neurons within the network in relation to network’s population burst. Specifically, CC quantitatively captures the answer to the following question: “With what frequency (i.e. how often) different proportions of constituting neurons participate in network’s population burst?” The *idealized case* when all constituting neurons participate in every population burst, CC takes the shape of CC_objective_ as depicted in Fig 2E.

Since our aim is to synchronize the bursting activities of network neurons, CC and CC_objective_ were chosen as the ‘characteristic curve’ and ‘desired characteristic’, respectively. Note that the extent of separation between CC corresponding to a given network activity and CC_objective_ provides an inverse measure of network synchronicity, i.e. the lesser the gap between CC and CC_objective_, the better the network synchronicity.

### 2.2 Handling network connectivity and network mutation

**Fig 3:**
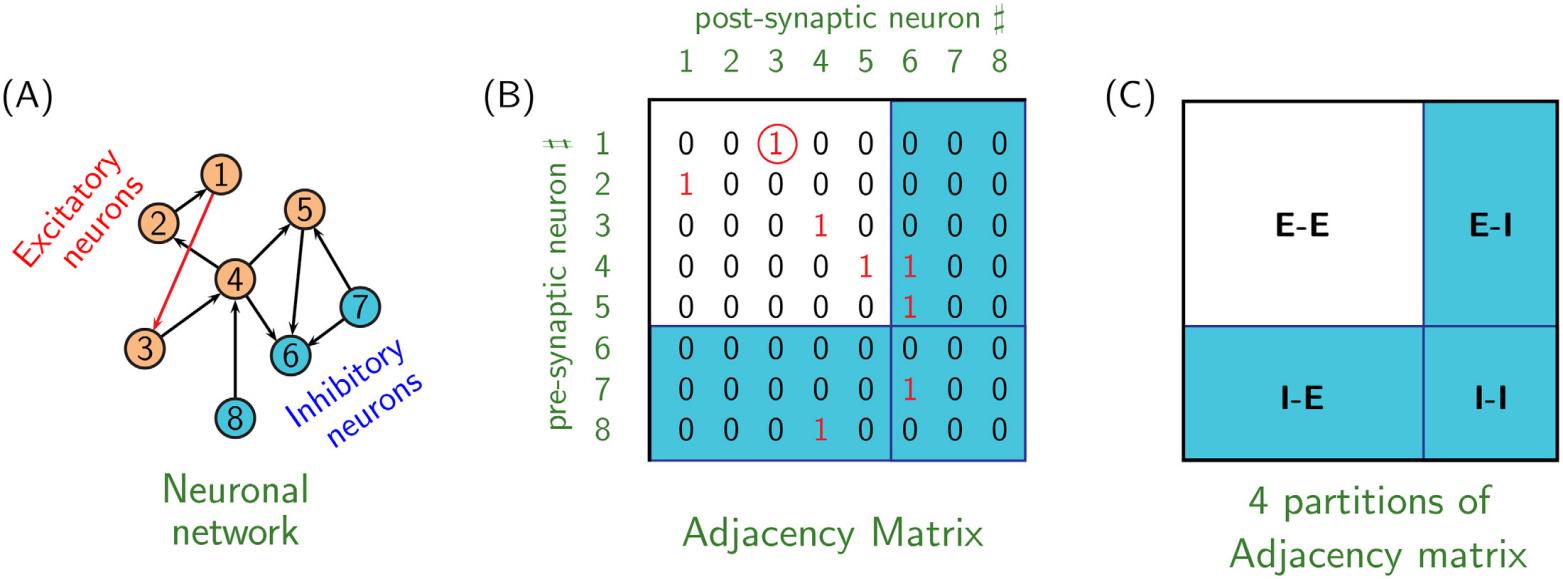
Figure illustrating usage of adjacency matrix for handling network connectivity. **(A)** Neuronal network consisting of 5 excitatory and inhibitory neurons, represented by tan and blue colors, respectively. **(B)** Adjacency matrix depicting the connectivity among neurons in network shown in (A). For example, red synapse connecting neuron 1 to neuron 3 in (A) is identified by presence of 1 at row 1 and column 3 in adjacency matrix. **(C)** Four partitions of adjacency matrix based on the nature of neuron, excitatory (represented by E) or inhibitory (represented by I) at its pre- and post-synaptic terminals. For example, partition E-I of adjacency matrix contains information of synapses emerging from excitatory neurons and terminating on inhibitory neurons.

The information of neuronal connectivity is handled in the form of ‘adjacency matrix’ [22]. If the neuronal group model is composed of *N* neurons, then size of adjacency matrix (also called connectivity matrix) is *N* × *N*. The presence of synaptic connection from *i*^*th*^ to *j*^*th*^ neuron within the neuronal group is stored as *a*_*ij*_ = 1, where *a*_*ij*_ is (*i*, *j*) element of adjacency matrix; otherwise *a*_*ij*_ = 0 (see Fig 3).

For mutating the network, using any of the four methods MM1-4 described in Appendix A, appropriate elements *a*_*ij*_ of adjacency matrix are selected and, switched from 0 to 1 and vice versa, to effect addition and deletion of synapses, respectively.

### 2.3 Developed evolutionary algorithm for evolving neuronal network topology

**Fig 4:**
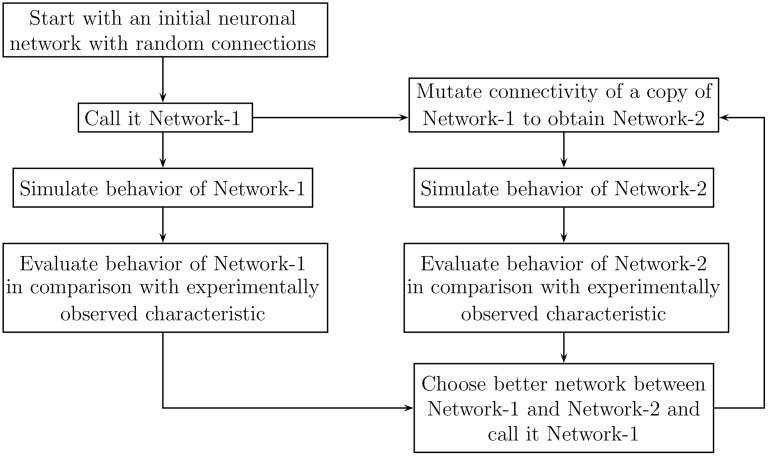
Simplified random hill optimization scheme used to evolve the network connectivity. The iterative evolution process is stopped when performance of mutated networks fails to improve for an appropriately set predefined number of consecutive iterations, which suggests optimal (or near-optimal) network evolution is achieved.

Two different algorithms were used for evolving the network:

1. **Random hill climbing:** A simplified scheme of random-hill climbing evolutionary algorithm utilized to evolve the network is outlined in Fig 4. In actual implementation, during the first iteration, 21 mutated copies (limited by number of usable processors in the SGI parallel computer, see section 2.7) of Network-1 are simulated in parallel. From them, 3 best performing networks (see sections 2.5 and 2.6 below) are chosen. Each selected network is used to produce 7 mutated networks for the next generation; thus, the 3 selected networks help to produce 21 mutated networks for the next generation. The iterative evolution process is stopped when performance of mutated networks fails to improve for 25 consecutive iterations, suggesting optimal (or near-optimal) network evolution is achieved. This evolutionary algorithm, in the present study, is used in conjunction with mutation methods MM1, MM2 and MM4 (see Appendix A), all of which preserve the total number of synapses present in the network.
2. **Simulated Annealing:** Note that the random-hill climbing algorithm described above has two important features: (1) the network continues to evolve ahead only if one of the newly mutated networks performs better than previous best network, and (2) mutation methods employed preserve the total number of synapses. In essence, the achievable evolutionary path by random hill climbing scheme is constrained by the fixed number of synapses used during network initialization. This restriction may be surpassed by altering the random-hill climbing algorithm in the following two ways: (1) at selection step choose the best networks from the newly mutated networks, regardless whether they perform better than the previously obtained best network or not, and (2) use an alternative mutation method MM3 that does not preserve the total number of synapses.
  The form of simulated annealing algorithm used for present study uses this modified form of random hill climbing algorithm as its basis; the only additional modification incorporated is that it uses both MM2 (total synapse count preserving) and MM3 (total synapse count non-preserving) to mutate the network (some of the next generation networks are obtained by application of MM2 while others by MM3). However, the essence of simulated annealing algorithm lies in its judicious use to selectively employ only one of MM3(SynAdd) and MM3(SynDel), in isolation, or both (but nevertheless always in conjunction with MM2) for evolving the network further at various stages of its evolution. In the present work, this selection is done *automatically* (See Appendix C).
  Additional features employed in Simulated Annealing algorithm:
  - As stated, during the selection step, the best networks from the newly mutated networks are chosen for further evolution, regardless whether they perform better than previously obtained best network or not. However, in case when there are two networks giving identical performance, then the network with lower total number of synapses is preferentially selected. This ensures that the network connectivity of the selected network has less redundant synapses, and is, therefore, more information rich regarding the network connectivity necessary to exhibit behavior corresponding to the ‘desired characteristic’.
  - Using MM2 and MM3 in conjunction in simulated annealing algorithm offers advantage in aforementioned sense, especially while only MM3(SynAdd) subtype of MM3 is employed. If any of the new network generated by MM2 performs as good as any other network generated by use of MM3(SynAdd), then the former network gets preferentially selected on account of its lower total synapse count.

In the present study, evolution study of two kind of neuronal networks were conducted: (1) network comprising solely of excitatory neurons (referred to as ‘excitatory network’ henceforth), and (2) network comprising of both excitatory and inhibitory neurons (referred to as ‘composite network’ henceforth).

Random hill climbing is one of the more prevalent methods employed in evolutionary algorithm paradigm. This was the first method that we experimented with for evolving the networks. We found that random hill climbing is sufficiently effective for evolving ‘excitatory network’. However, we faced difficulties in evolving the ‘composite network’ with it, and hence subsequently devised the above-described version of simulated annealing algorithm.

### 2.4 Allocation of neuronal properties

**Fig 5:**
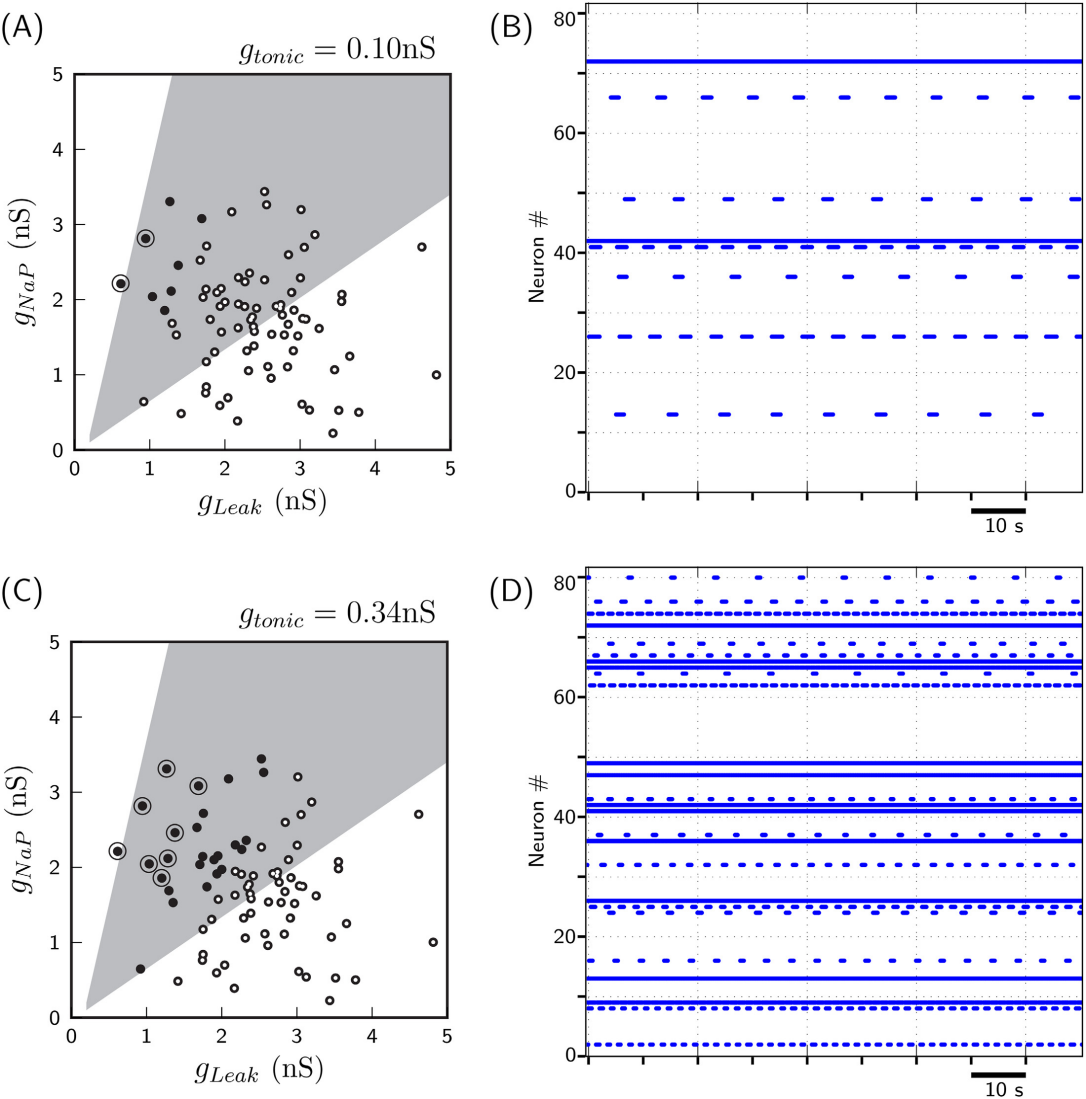
Allocation of neuronal property and neuron’s ‘isolated’ behavior. **A&B for** *g*_*tonic*_ = 0.10**nS, and C&D for** *g*_*tonic*_ = 0.34**nS**. A: Distribution of 80 neurons constituting the neuronal network over *g*_*Leak*_/*g*_*NaP*_-parametric space. B: Raster plots depicting activities of *synaptically isolated* 80 neurons depicted in A. Neurons exhibiting periodic ‘bursting’, ‘tonic’ and ‘silent’ activity in B are indicated as solid, circled and open dots, respectively, in A. Note that A&B correspond to the case when *g*_*tonic*_ = 0.10nS for neurons. C&D provide analogous plots for the case when *g*_*tonic*_ = 0.34nS for neurons.

In the present study, we use neuronal model developed by Butera et al. [a modified version of *model 1* in [23] as described in [4]]. In this model, bursting and non-bursting behavior of neurons are determined by their leak conductance *g*_*Leak*_ and persistent sodium conductance *g*_*NaP*_. It is assumed that distribution of *g*_*Leak*_ and *g*_*NaP*_ values among neurons conforms to two dimensional Gaussian distribution. Thus, allocation of *g*_*Leak*_ and *g*_*NaP*_ values among neurons was done by generating random numbers having two-dimensional Gaussian distribution with *g*_*Leak*(*mean*)_ = 2.51 nS and *g*_*NaP*(*mean*)_ = 1.8 nS; standard deviation was assumed to be 1.47 nS in either direction. Values of *g*_*Leak*_ and *g*_*NaP*_ deviating from the mean value by greater than 2.5 nS were discarded (so as to avoid allocating negative values of *g*_*Leak*_ and *g*_*NaP*_). A typical distribution of allocated neuron property (80 neurons) constituting the neuronal network is depicted in Fig 5A. Intrinsic bursting frequencies of various neurons and network’s intrinsic interburst intervals are presented in S1 Text.

#### 2.4.1 Pacemaker/non-pacemaker neurons and Isolated neuronal behavior

The shaded region in Fig 5A depicts the range of values of (*g*_*Leak*_, *g*_*NaP*_) for which a neuron possesses pacemaking property i.e. for low enough value of *g*_*tonic*_ neurons in this region remain silent, and as the *g*_*tonic*_ value is progressively increased, they exhibit ‘bursting’ behavior for some range of *g*_*tonic*_ before transiting to ‘tonic’ behavior at higher values of *g*_*tonic*_ [4]. Neurons in unshaded regions are non-pacemaker neurons; they transit directly from silent to tonic behavior with increase in *g*_*tonic*_ value, and do not exhibit bursting behavior for any range of *g*_*tonic*_. Note that the classification of a neuron as pacemaker or non-pacemaker is completely determined by the pair (*g*_*Leak*_, *g*_*NaP*_); value of *g*_*tonic*_ is free to vary.

However, what behavior a *synaptically isolated* neuron exhibits is determined by the triplet (*g*_*Leak*_, *g*_*NaP*_, *g*_*tonic*_). For a set value of *g*_*tonic*_, a neuron may exhibit three different behaviors: tonic, bursting or silent. For instance, for the set value of *g*_*tonic*_ = 0.10nS for all 80 neurons depicted in Fig 5A, two exhibit tonic behavior, 6 exhibit bursting and the rest are silent; see Fig 5B. Note that both pacemaker and non-pacemaker neurons may exhibit tonic firing or may remain silent; however, only pacemaker neurons may exhibit bursting.

### 2.5 Evolving excitatory network

We constructed a neuronal network comprising of 80 excitatory neurons whose neuronal properties were allocated as depicted in Fig 5A. Note that only 10–15% of them are intrinsically active (bursting/tonic), see Fig 5A and 5B corresponding to *g*_*tonic*_ = 0.10nS. The network connectivity was randomly initialized with 10% of all-to-all connectivity (for short, *SynFrac* = 0.10); the rationale for choosing sparsely connected network is discussed in section 4.3. The synaptic strength (henceforth, *SynStrength* for short) was chosen low enough (0.48nS) so that the initial random network could barely exhibit population burst; we adopted the criteria that when more than 30% network neurons exhibit bursting simultaneously, it constitutes a population burst. The initialized random network is first evolved using the random hill climbing algorithm, with MM1 as mutation method, and subsequently, by using the simulated annealing algorithm, towards the dual objectives:

**Objective-EN1:** Evolved network produces better synchronized population bursts. Criteria used: A network in which higher fraction of neurons participate in each population burst is a better synchronized network.
**Objective-EN2:** Network produces regular population bursts at a predefined frequency. In the present study, desired inter-burst interval (IBI) was set to 7s (arbitrarily).

The degree of synchronization of a network is quantitatively obtained by computing the cumulative curves (CC) as illustrated in Fig 2. As per our adopted criteria, a network in which all neurons participate in every population burst is the best synchronized network (Fig 2E); cumulative curve corresponding to such a network is indicated by CC_objective_ in Fig 2F. The extent of separation between CC corresponding to an arbitrary network with that of CC_objective_ provides an inverse measure of network synchronicity. This is quantitatively measured as

$$\begin{array}{c} \text{Cost}1=\alpha \int_{0}^{1}{\left({\text{CC}}_{\text{network}}-{\text{CC}}_{\text{objective}}\right)}^{2}dx=\alpha \sum_{j}{\left({\text{CC}}_{\text{network}}-{\text{CC}}_{\text{objective}}\right)}_{j}^{2}\Delta x_{j} \end{array}$$ (1)

where *α* is a scaling factor, and subscript *j* represents the index of sub-intervals Δ*x*_*j*_ in which range [0, 1] of CC is subdivided into. To quantify the closeness of the network with respect to Objective-2, the following measure was used

$$\begin{array}{ccc} \text{Cost}2 & = & \beta \times {\left({\text{IBI}}_{\text{network}}-{\text{IBI}}_{\text{objective}}\right)}^{2} \end{array}$$ (2)

where *β* is a scaling factor, and IBI_objective_ = 7s. Thus, for evolving the excitatory network towards dual objectives EN1 and EN2, Total Cost = (Cost1 + Cost2) was minimized. We empirically set scaling factors *α* = 50 and *β* = 1.0. The value of all sub-intervals Δ*x*_*j*_ while computing Cost1 using Eq (1) was set to 0.02. The motivation behind choice of objective functions and scaling factor values is discussed in section 4.1.

### 2.6 Evolving composite network

We constructed a neuronal network, comprising of 160 neurons—80 excitatory and 80 inhibitory neurons. The neuronal properties of both 80 excitatory and 80 inhibitory neurons were allocated as depicted in Fig 5C. Note that the value of *g*_*tonic*_ for both excitatory and inhibitory neurons, in comparison to 0.10nS in Fig 5A, was set to a higher value of 0.34nS. This was done to increase the degree of sporadic bursts among excitatory neurons. Here, every neuronal spike which occurs during non-occurrence of network’s population burst and is not a part of pre- or post-inspiratory phase of any neuronal burst is considered to constitute sporadic burst. A total of 26 of 80 neurons possess intrinsic bursting/tonic property at this value of *g*_*tonic*_ (Fig 5C and 5D). The network connectivity was randomly initialized with 6% of all-to-all connectivity (*SynFrac* = 0.06), with excitatory and inhibitory *SynStrength* values set to 0.6nS and 0.36nS, respectively. Using the simulated annealing algorithm, as described in section 2.3, we evolved a randomly initialized network towards the following three objectives:

**Objective-CN1:** Evolved network produces better synchronized population bursts. Criteria used: A network in which higher fraction of *excitatory* neurons participate in each population burst is a better synchronized network.
**Objective-CN2:** Identical with objective-EN2 stated in section 2.5.
**Objective-CN3:** Sporadic bursts of *excitatory* neurons is minimum. This is perhaps effected by inhibitory neurons by providing IPSP to excitatory neurons.

The motivation behind choice of objectives is discussed in section 4.1. Note that the contribution of inhibitory neurons to network synchronization (i.e. to Objective-CN1) is assumed to be indirect; they may potentially influence the activity of excitatory neurons by providing IPSPs, which may help match the onset of bursting of excitatory neurons and, thus, to exhibit synchronized population burst.

The costs associated with first two objectives (CN1 andCN2) are quantified using Eqs (1) and (2). The cost associated with Objective-CN3 is quantified as

$$\begin{array}{ccc} \text{Cost}3 & = & 100\times \frac{\text{total sporadic burst count}}{\text{total sporadic burst count possible}} \end{array}$$ (3)

The convention employed for computation of ‘total sporadic burst count’ and ‘total sporadic burst count possible’ is described in Appendix B. Thus, for evolving the network, the aim is to minimize the Total Cost = (Cost1 + Cost2 + Cost3). This is carried out by employing the simulated annealing algorithm as described in section 2.3. See Appendix C for details.

### 2.7 Simulation Overview

Simulations were done using ISM parallel supercomputers (PRIMERGY RX200S5 x 360, 200 x 8 cores, Fujitsu, Japan, and SGI ICE X, 400 nodes, SGI, USA). For evolving both ‘excitatory network’ and ‘composite network’, during each iteration the newly mutated networks were simulated for 180,000ms (equivalent to 180s of real neuronal time) with a time step size of 0.05ms using forth order Runge-Kutta method in C++. To discount the initial transients, first 40s of real neuronal time were discarded. Data saved for the time range 40–180s (with a bin-width of 160ms) were used to evaluate performance of networks against the set objectives described in previous two sections. Note that the chosen bin-width of 160ms is larger than the largest inter-spike interval belonging to the same neuronal burst and smaller than typical interburst interval; thus it is adequate to identify the beginning and end-timings of individual neuronal bursts. The data thus saved is adequate for computations of Cost1, Cost2 and Cost3 besides keeping the saved data to manageable size.

In real time, simulation duration for evolving ‘excitatory network’ amounts to ≈3.6min per iteration. Since ‘composite network’ consists of twice as many neurons as in ‘excitatory network’, simulation duration for its evolution is ≈7.2min per iteration.

## 3 Results

### 3.1 Evolved ‘excitatory network’ towards improved synchronicity

**Fig 6:**
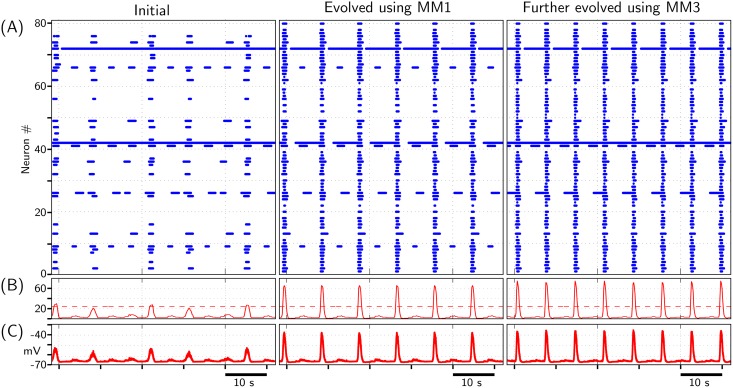
Network activity before and after evolution using MM1 and, subsequently using MM3. Network is evolved to minimize (Cost1+Cost2) as defined using Eqs (1) and (2). (A)‘Initial’: Raster plot depicting activities of 80 neurons depicted in Fig 5A when they are randomly synaptically connected with *SynFrac* = 0.10. (A)‘Evolved using MM1’: Raster plot depicting activities of same 80 neurons when the network connectivity is evolved to optimum using MM1; *SynFrac* is maintained fixed at 0.10. This evolved network is then further evolved using MM3 and its performance is shown in (A)‘Further evolved using MM3’. (B) Solid curve: Time-history of total number of bursting neurons at a given instant. Dashed horizontal line: Threshold level, set at 30% of 80 neurons (our adopted convention), which when exceeded by ‘solid curve’, the network is considered to be exhibiting a population burst. (C) Population activity (averaged membrane potential) of all 80 neurons.

The evolutionary change in the activity of excitatory network as it evolves from randomly initialized network to a better synchronized network following the method described in section 2.5 is depicted in Fig 6. The network is initially evolved using the random hill climbing algorithm, with MM1 as mutation method, and subsequently, by using simulated annealing. For evolving the network towards dual objectives-EN1 and EN2, Total Cost = (Cost1+Cost2) was minimized. Successive improvements in network synchronization were observed during the evolutionary process, beginning from initial random network to eventual evolved network (S8 Fig). The gap between CC_MM1_ and CC_objective_ reflects a limit to the maximum optimization that may be achieved by ‘constrained’ mutation method MM1; ‘constrained’ in the sense that MM1 attempts to optimize network’s performance by keeping *SynFrac* value *fixed*. This is corroborated by further evolution of network using MM3 (See S7 and S8 Figs). Though the results depicted in Fig 6 are obtained using the random hill algorithm with MM1 as mutation method (and subsequently, MM3), usage of MM2 instead of MM1 produces similar results as well. In fact, the optimized cumulative curve using MM2 is almost identical to that using MM1 in S8 Fig.

#### 3.1.1 Skewed/Heavy-tailed degree distribution within evolved network

**Fig 7:**
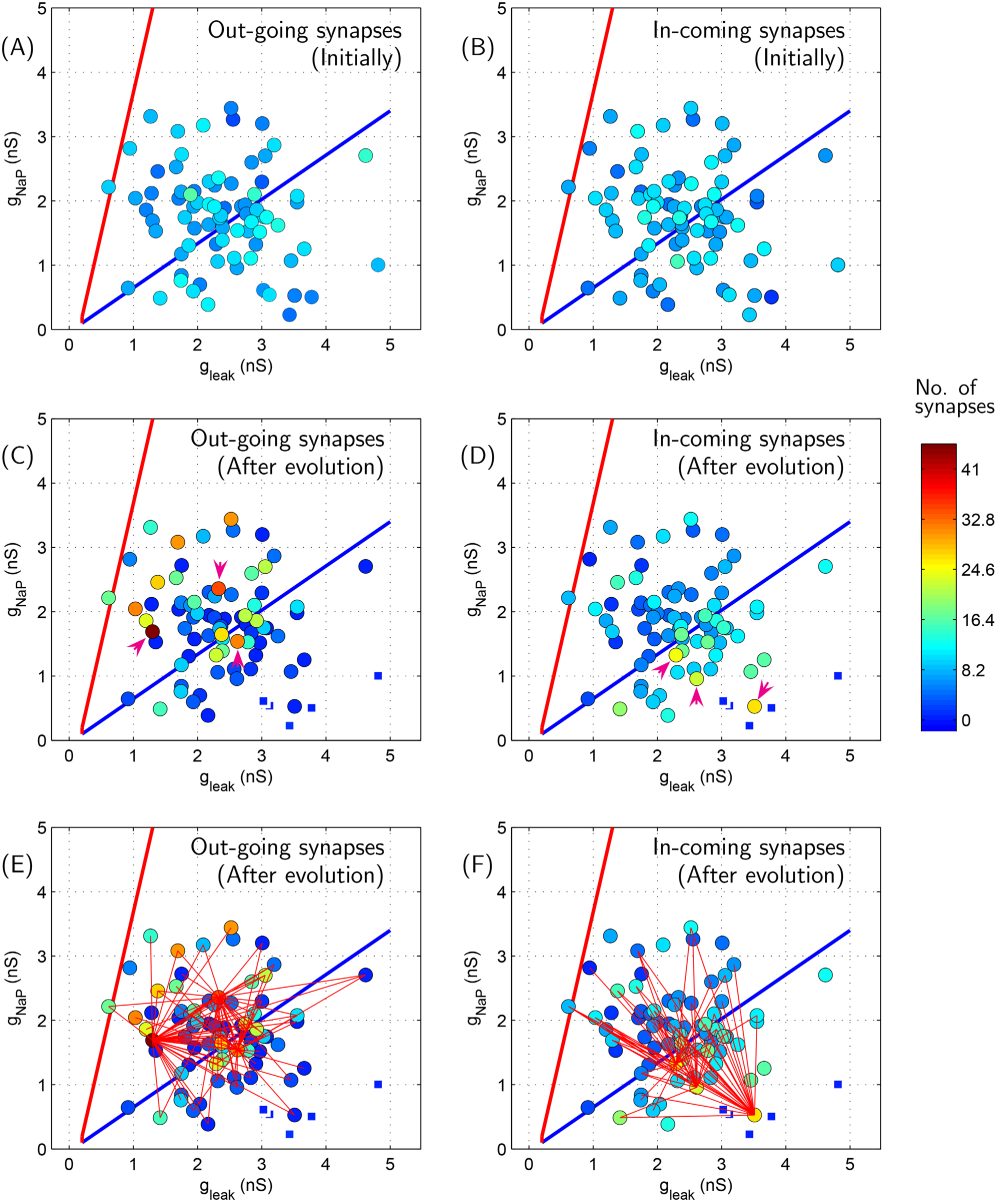
Synaptic distribution among network neurons before and after evolution (using MM3) corresponding to the case presented in Fig 6. (A) Initial incoming synaptic distribution, (B) Initial outgoing synaptic distribution, (C) Outgoing synaptic distribution after evolution, and (D) Incoming synaptic distribution after evolution. Neurons indicated in square shape with thick white borders (consequently, appearing smaller in size) towards the bottom-right region in each panel are those which remain silent in the ‘evolved’ network activity, Fig 6. (E)&(F) Connectivity of top three richest neurons in (C)&(D), respectively, with respect to rest of the neurons in the network. The top three richest neurons in (C)&(D) are indicated by magenta colored arrows for convenience.

**Fig 8:**
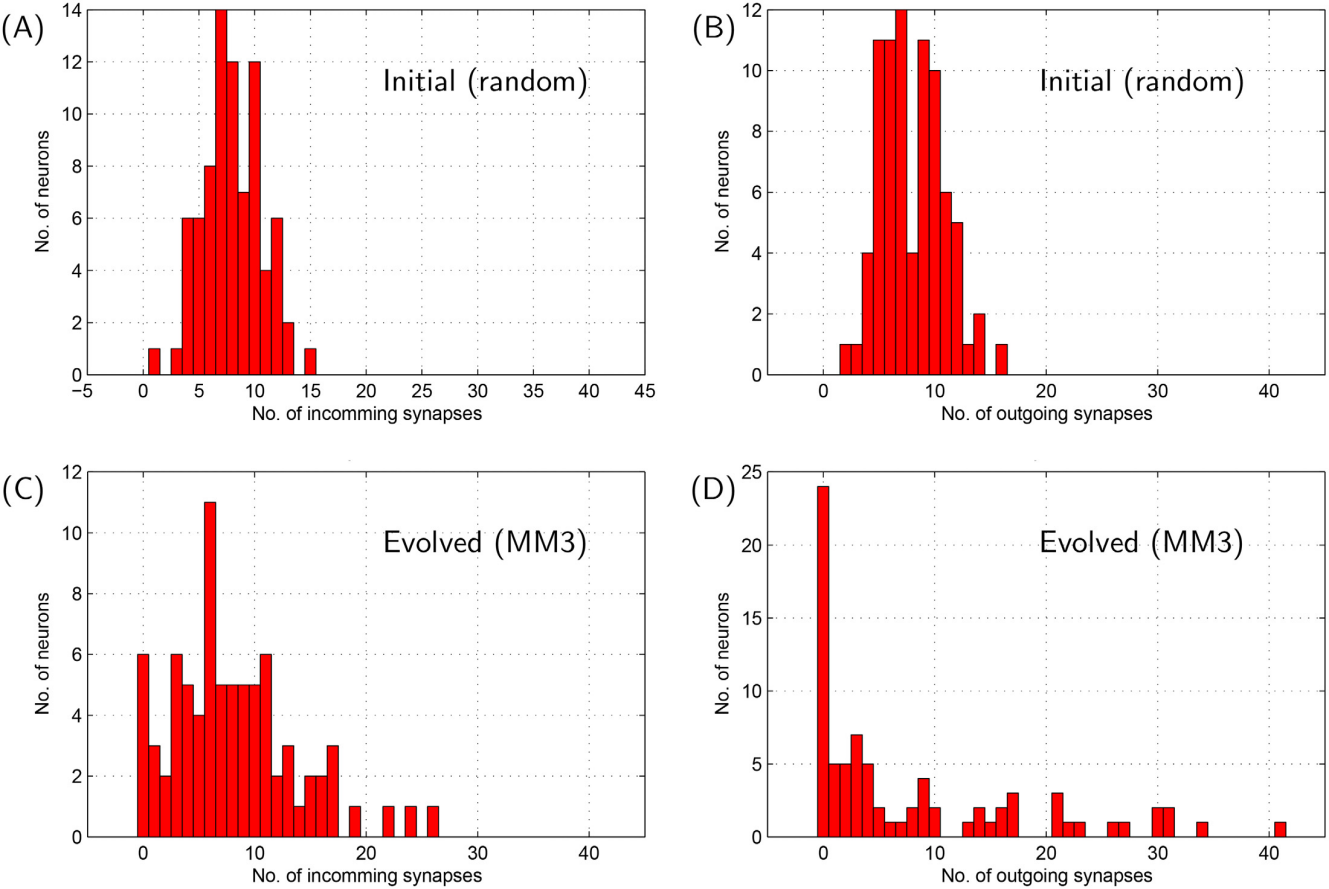
Histograms indicating distribution of neurons with various degrees of out-going and incoming synapses in the evolved network depicted in Fig 7.

The synaptic distribution among the network neurons before and after network evolution is depicted in Figs 7 and 8. As may be readily observed, the initial relatively homogenous synaptic distribution becomes extremely heterogenous after evolution. Fig 8C and 8D suggests a tendency of distributing increasingly large number of out-going and in-coming synapses among very few neurons within the evolved network.

Further, Fig 7C and 7D suggests that in the evolved network, neurons rich in out-going synapses are mostly pacemaker neurons while less readily excitable non-pacemaker cells are rich in in-coming synapses. Moreover, connectivity pattern depicted in Fig 7E and 7F suggests that the evolved network is rich with synapses having pacemaker and non-pacemaker neurons at pre- and post-synaptic ends, respectively.

#### 3.1.2 Comparing the performance of evolved ‘excitatory network’ with that of Small-world network

As mentioned in the Introduction section, small-world (SW) network is one of the most common network studied in context of network synchronization phenomena [20], and is often suggested as a possible network structure within the preBötC [18]. We compared the level of synchronization that may be achieved by small-world (SW) network with that achievable by evolved excitatory network.

First we constructed a SW network with 10% of all-to-all connectivity (moderately sparse) using Watts and Strogatz model [20]. Specifically, first all 80 neurons are assumed to be aligned along the circumference of a circle and from each neuron exactly 8 synapses are extended—one each to 4 neurons on its left and 4 neurons on its right. Following this, 10% of the synapses of the regular network so obtained was randomly repositioned (keeping the pre-synaptic end fixed, only the post-synaptic end of synapse is randomly repositioned). Self connectivity was avoided during the initialization of SW network.

**Fig 9:**
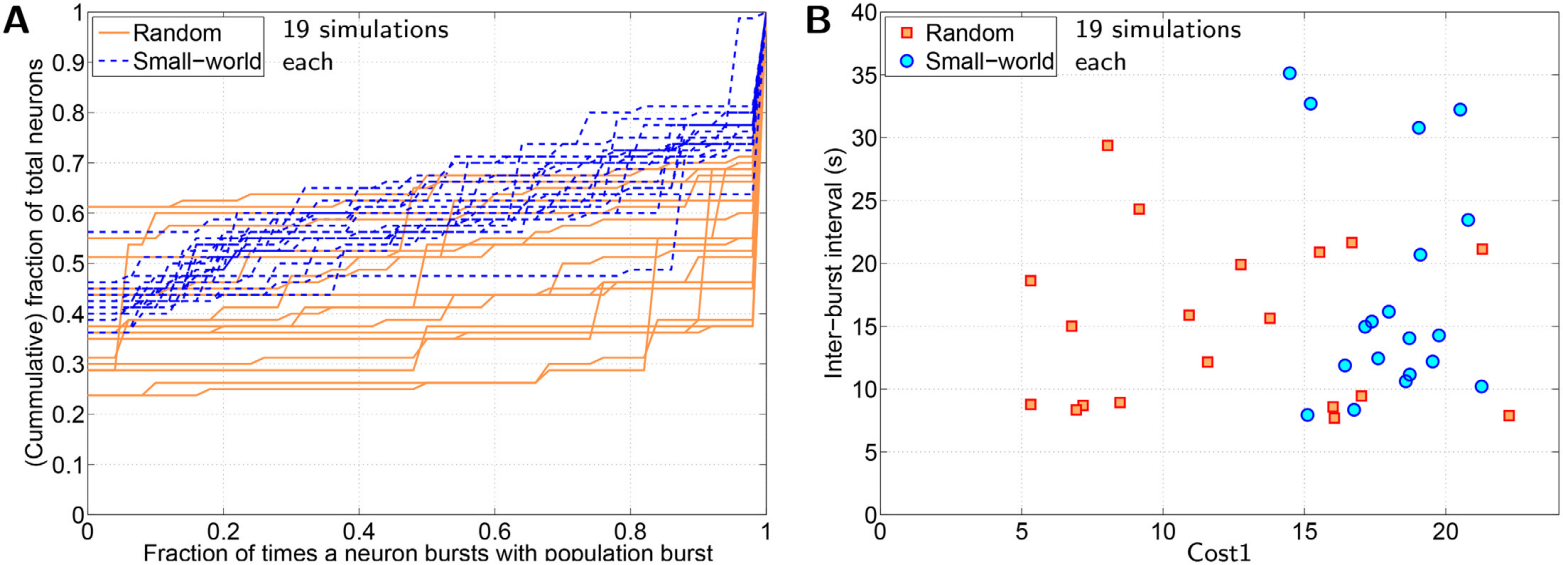
Comparison between random network and small-world (SW) network. A: Cumulative curves corresponding to neuronal activity obtained for a set of 19 randomly initialized network compared against those obtained for a set of 19 different SW networks. Allocated neuron properties for constituent network neurons, synaptic density (10% of all-to-all connectivity) and synaptic strength are identical for all networks. B: Cost1 vs IBI (inter-burst interval) scatter plot of 19 pairs of random and SW networks in A.

The performance of 19 different SW networks against those of 19 different randomly initialized network (with 10% of all-to-all connectivity, same as that for SW networks) is depicted in Fig 9. It is evident that the SW networks is no better at synchronizing the neuronal network than random networks. Note that *SynStrength* for the comparison shown in Fig 9 was set to 0.64nS, which was greater than the *SynStrength* at which the network evolution in Fig 6 was carried out (0.48nS). This is because for *SynStrength* = 0.48nS, SW network fail to exhibit synchronized population burst; none of the 21 initialized SW networks exhibit synchronized population burst as opposed to 5–8 of 21 randomly initialized network.

**Fig 10:**
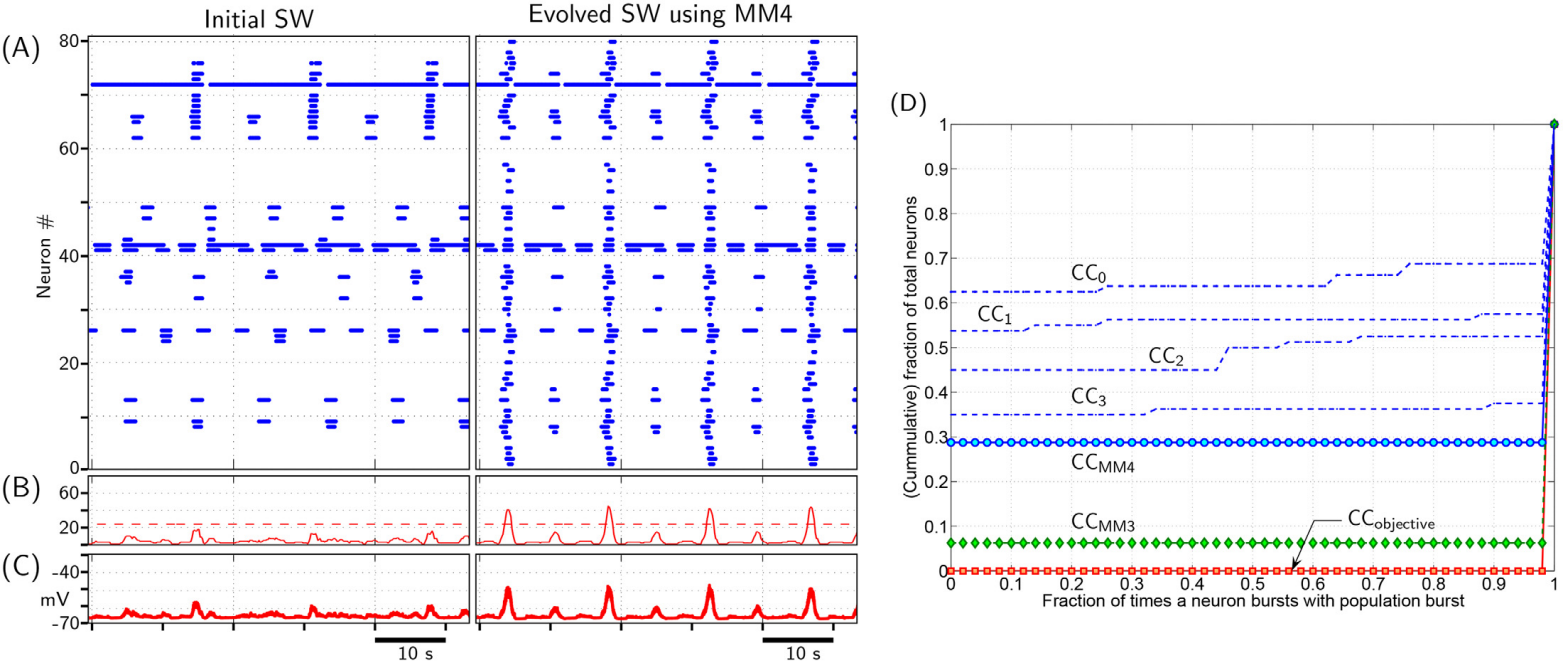
Evolution of small-world (SW) network. (A,B & C) Typical neuronal activity of a SW network, initially and after evolving it using MM4. Objective of evolution is to minimize (Cost1+Cost2) as defined by Eqs (1) and (2), respectively. (D) Evolution of cumulative curves (CC) depicting participation level of individual neurons to each population burst. CC_objective_: CC corresponding to the desired case when every neuron in the network bursts with every population burst. CC_MM4_ is CC corresponding to network activity depicted in (A)‘Evolved SW using MM4’. CC_0_, CC_1_, CC_2_ and CC_3_ are representative intermediate CC obtained during the evolution process. CC_MM3_ is CC corresponding to evolved excitatory network by method described in section 2.5 (it is the same CC_MM3_ curve as is depicted in S8 Fig).

Following this, the random hill climbing algorithm using MM4 as mutation method was used to evolve a SW network with the objective of improving the network synchronicity. Note that MM4 preserves the initial SW network topology intact; performance of neuronal network improves as a consequence of neurons swapping positions within the network. Fig 10 indicates neuronal activity of a typically evolved SW network using MM4. Since CC_MM4_ is farther away from CC_objective_, as compared to CC_MM3_, it may be concluded that even the best evolved SW network provides suboptimal synchronization of neuronal network. Additional result supporting this conclusion is provided in S2 Text.

### 3.2 Evolution of ‘composite network’

**Fig 11:**
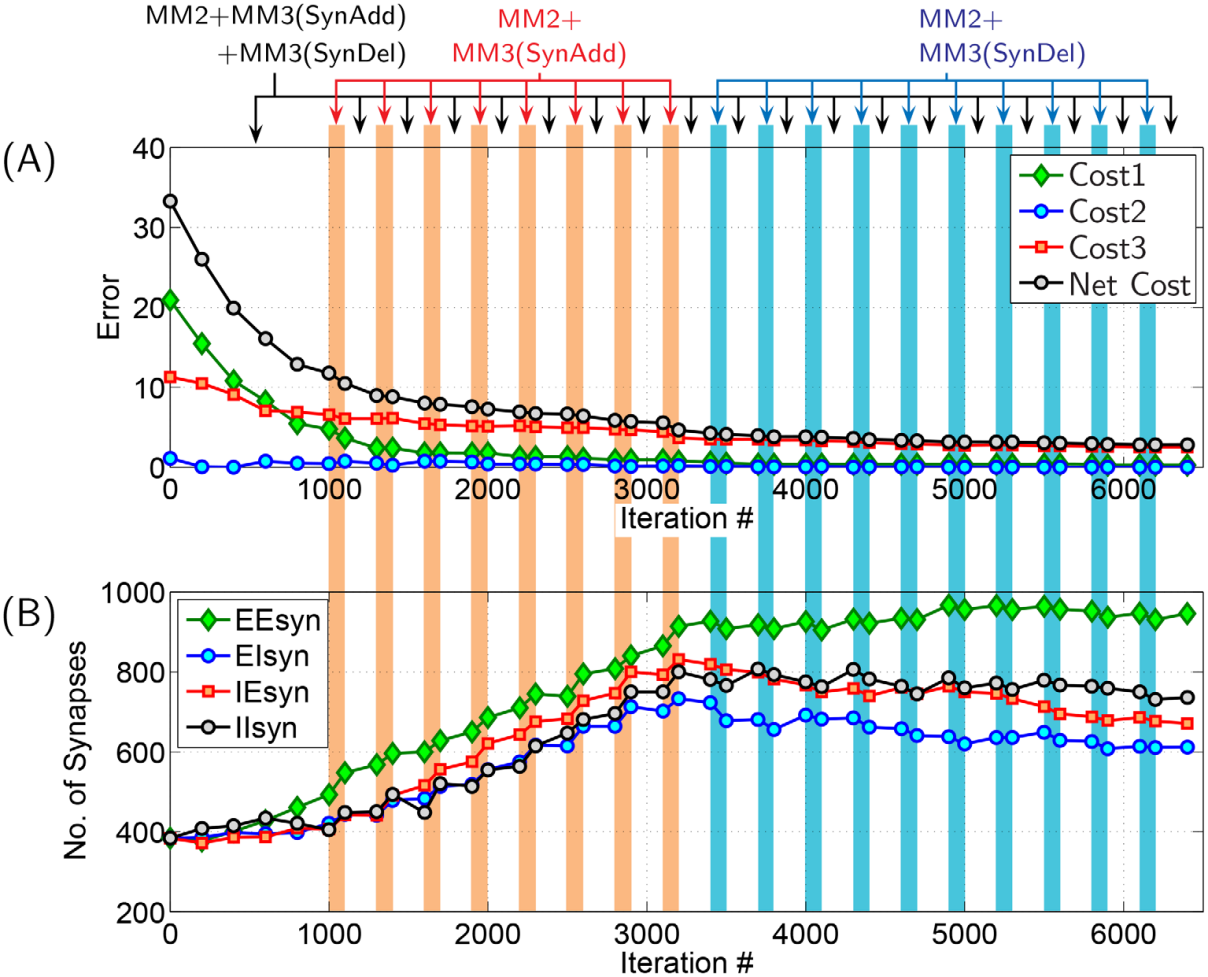
Time-history of composite network evolution. **(A)** Depiction of evolution of network in terms of ‘Cost’ minimization. Cost1, Cost2 and Cost3 are given by Eqs (1), (2) and (3), respectively. For network evolution, objective is to minimize (Cost1+Cost2+Cost3). Vertical color stripes indicates mutation methods that were employed during various stages for evolving the network. For further explanation see Appendix C. **(B)** Variation of synapses within the network during evolution process. Nomenclature: EEsyn, EIsyn, IEsyn and IIsyn are total synapses within EE, EI, IE and II part of adjacency matrix, see Fig 3C.

**Fig 12:**
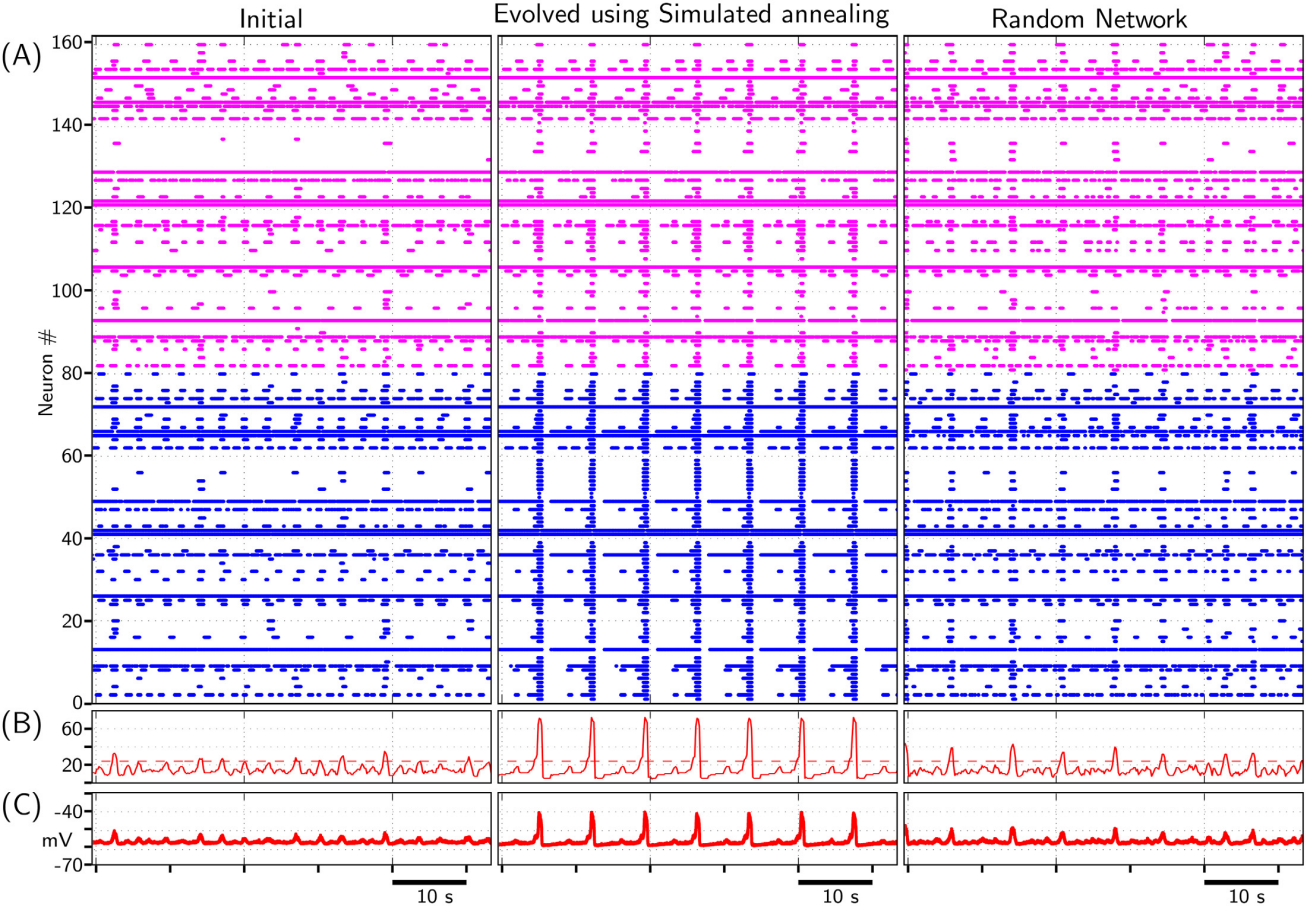
Composite network activity before and after evolution using MM3. Network is evolved to minimize the cost (Cost1+Cost2+Cost3), see Fig 11. (A)‘Initial’: Raster plot depicting activities of 160 neurons—first 80 are excitatory neurons while the rest are inhibitory neurons—within the neuronal network when they are randomly synaptically connected with *SynFrac* = 0.06; (A)‘Evolved using Simulated annealing’: Raster plot depicting activities of same 160 neurons when the network connectivity is evolved using simulated annealing. *SynFrac* value varies during evolution process (see Fig 11); final *SynFrac* ≈ 0.12. (A)‘Random Network’: Raster plot depicting activities of 160 neurons in a random network with *SynFrac* ≈ 0.12. (B) Solid curve: Time-history of total number of bursting neurons at a given instant. Dashed horizontal line: Threshold level, 30% of 80 excitatory neurons (our adopted convention), which when exceeded by ‘solid curve’, the network is considered to be exhibiting a population burst. (C) Population activity (averaged membrane potential) of all 80 excitatory neurons.

Evolution of composite network using simulated annealing algorithm in terms of ‘Cost’ minimization along with variation in synapse count within the network is depicted in Fig 11. *SynFrac* value in the evolved network is ≈0.12, which is greater than *SynFrac* = 0.06 with which the network was randomly initialized. Fig 12 depicts the ‘Initial’ and ‘Evolved’ population activity of all 160 neurons within the network. Though the strengths of excitatory and inhibitory synapses are fixed a priori and remain unchanged all through network evolution,the synaptic count within the network changes—the ‘Initial’ network is randomly connected with *SynFrac* = 0.06, while the ‘Evolved’ network has a *SynFrac* = 0.12. Thus, to compare its performance, a random network with *SynFrac* = 0.12 was constructed and simulated; its performance is depicted in rightmost column of Fig 12. The evolved network is synchronized better than the random network (with the same *SynFrac*) when measured against the objectives-CN1, CN2 and CN3 stated above. Elimination of IPSPs in the evolved network 1) decreased IBI of network from 7.1s to 6.64s, 2) increased sporadic bursting of excitatory neurons, and 3) activity of inhibitory neurons got better synchronized with each population burst (see S11 Fig).

#### 3.2.1 Characteristics of synaptic connections in the evolved composite network

**Fig 13:**
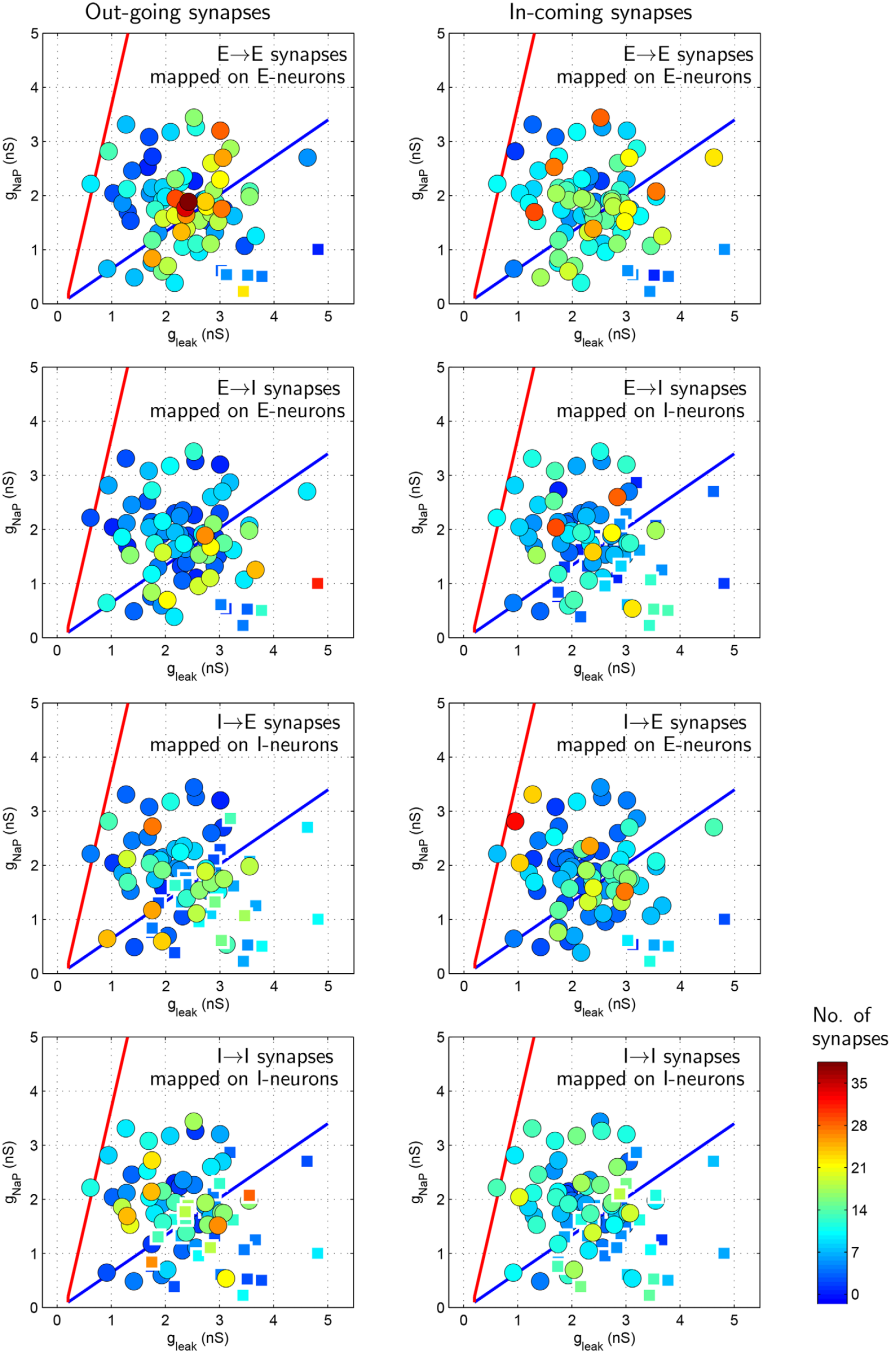
Synaptic distribution among neurons of the evolved network corresponding to the case presented in Fig 12. Neurons indicated in square shape with thick white borders (and consequently, appearing smaller and fainter) in each panel are those which remain silent in the ‘evolved’ network activity, Fig 12. Initial synaptic distribution among neurons is randomly distributed with overall *SynFrac* = 0.06, which is lower than that of evolved network (*SynFrac* ≈ 0.12) and hence is not shown.

**Fig 14:**
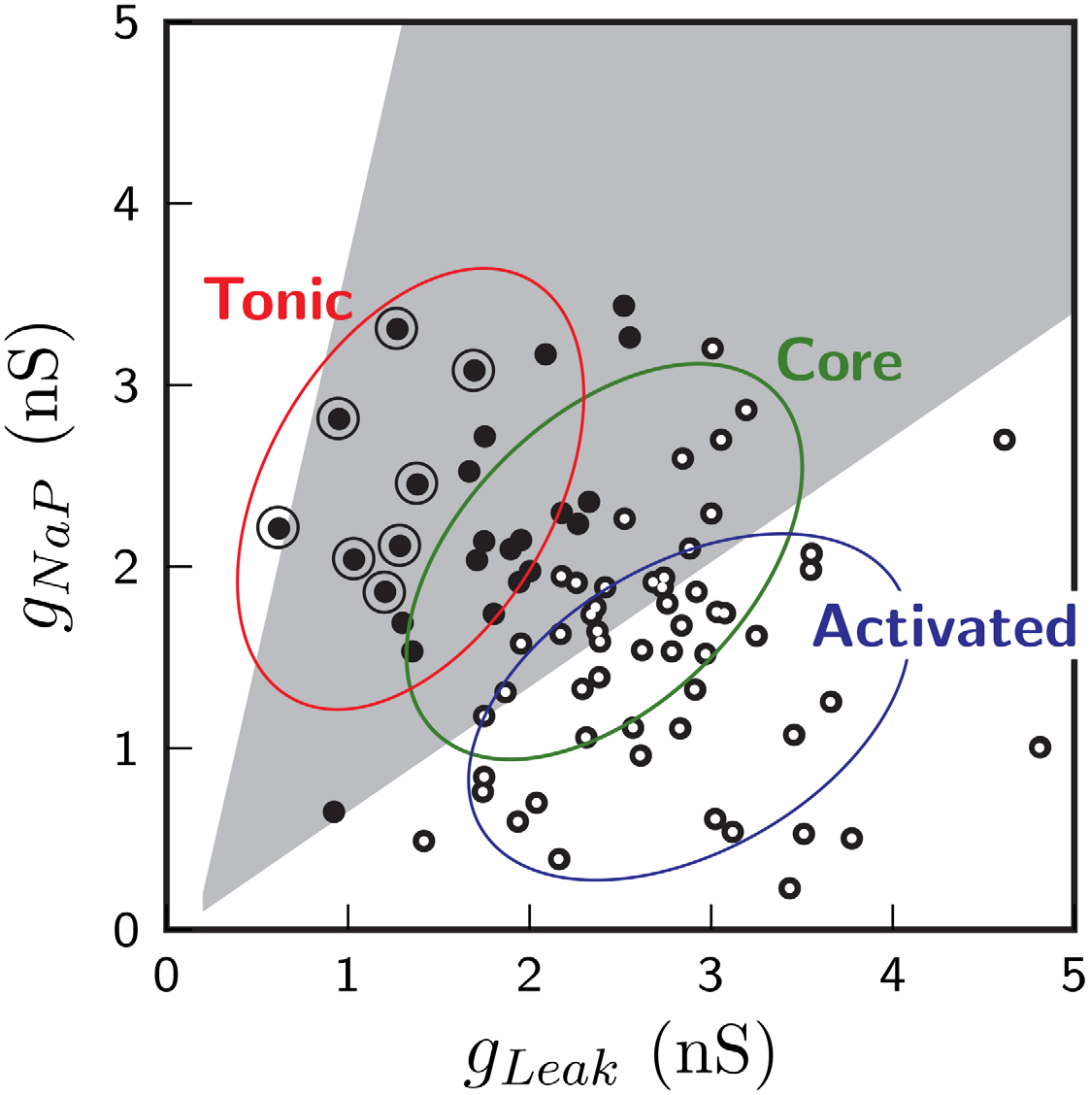
Classification of *g*_*Leak*_/*g*_*NaP*_-parametric space into three regions—Tonic, Core and Activated. Distribution of 80 neurons constituting the neuronal network over *g*_*Leak*_/*g*_*NaP*_-parametric space is also indicated and is identical with that depicted in Fig 5C. See section 3.2.1 for further explanation.

A typical synaptic distribution among neurons of the evolved network is depicted in Fig 13. In order to characterize the synaptic distribution presented in Fig 13, we partitioned the *g*_*Leak*_/*g*_*NaP*_-parametric space into three distinct regions (see Fig 14):

- ‘Tonic region’ comprises neurons which exhibit tonic behavior in synaptically isolated state (see section 2.4.1). These neurons tend to exhibit tonic behavior even when synaptically connected, within the neuronal networks.
- ‘Core region’ primarily comprises non-tonic pacemakers neurons i.e. pacemaker neurons which do not exhibit tonic behavior in synaptically isolated state. Within neuronal network, they respond *readily* to EPSPs they receive from other neurons to exhibit bursting.
- ‘Activated region’ neurons are intrinsically silent non-pacemaking neurons which become active in the network as a consequence of EPSPs they receive from other neurons. The level of EPSPs required to make these neurons active is greater than that necessary for exciting ‘core region’ neurons.

The intrinsic excitability of neurons is highest in the ‘tonic region’, intermediate for ‘core region’ and lowest in the ‘activated region’. However, since the excitability of neurons within these regions varies gradually, the region boundaries are not strict, but slightly overlapping. Note that both excitatory and inhibitory group of neurons of composite network may be subclassified into ‘tonic region’, ‘core region’ and ‘activated region’ neurons. Thus, composite network neurons may be categorized into ‘excitatory tonic region’ neuron, ‘inhibitory core region’ neuron, and so forth.

**Fig 15:**
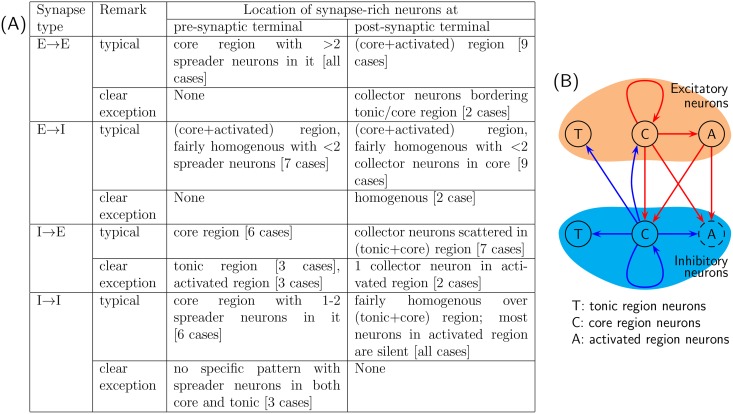
Summary of synaptic distribution observed in evolved network. **(A)** Summary of general characteristics, labeled as ‘typical’, of synaptic distribution observed in evolved network in 12 different simulations. Results of certain simulations unambiguously differed from ‘typical pattern’; these pattern are labeled as ‘clear exceptions’ and are listed as well. Pattern found in remaining simulations conformed to the ‘typical pattern’ with minor variations. **(B)** A visual representation of ‘typical connectivity pattern’ summarized in table (A), assuming that neurons rich with outgoing synapses predominantly connect to neurons rich with incoming synapses. Note that (B) indicates only predominant connectivity pattern among various kind of neurons; see section 3.2.1 for further explanation. Inhibitory ‘activated region’ neurons largely remain silent in the evolved network, and hence are represented by a dashed circle in (B).

Referring to Fig 13, it may be noted that the excitatory ‘core region’ neurons are typically rich in out-going synapses; in a way these neurons set the rate of network’s periodic burst. Fig 15A provides a summary of general characteristics of synaptic distribution observed in the evolved network. Neurons with large number of incoming synapses and outgoing synapses are termed as ‘collectors’ and ‘spreaders’, respectively, in Fig 15A. From this table, it is concluded that the most common feature of the idealized network is to have ‘spreader’ neurons within both excitatory and inhibitory neuronal network. Under the simplistic assumption that neurons rich with outgoing synapses predominantly connect to neurons rich with incoming synapses, a visual summary of ‘typical connectivity pattern’ in Fig 15A is depicted in Fig 15B. Note that Fig 15B indicates only predominant connectivity pattern among various kind of neurons; in general, there are some random synapses interconnecting neurons from all region. For instance, excitatory neurons from tonic region do posses outgoing synapses to various neurons (see Fig 13). But since these synapses do not exhibit any predominant connectivity pattern, no out-going synapses from excitatory ‘tonic region’ neuron are depicted in Fig 15B. In addition, majority of inhibitory neurons belonging to ‘activated region’ tend to remain silent in the evolved network. Apparently, their presence in the evolved network is redundant.

All 12 evolved composite networks for which the results are summarised in Fig 15, excitatory and inhibitory *SynStrength* values are set to 0.6nS and 0.36nS, respectively. To check whether results are robust against variation in *SynStrength* values, additional simulations were carried out by varying excitatory and inhibitory *SynStrength* values in the range 0.2–0.6ns (incrementing in steps of 0.2nS) and 0.12–0.48nS (incrementing in steps of 0.12nS), respectively—9 cases in all. The reduction in *Synstrength* values lead to increase in total synapse count within the evolved network (see section 4.5) and vice versa; however, synaptic connectivity pattern within these additional evolved composite networks were found to be consistent with the pattern summarized in Fig 15.

## 4 Discussion

### 4.1 Remarks on Objective functions for network evolution

**Objective-CN1:** Objective-CN1 for evolving random networks used in the present study is an idealization towards improved synchronicity. Such behavior is typically not experimentally observed in preBötC slices. However, it serves as an idealization of the functional purpose of excitatory network within the preBötC. According to current understanding [2, 24, 25], it is the excitatory network within preBötC that enables rhythm generation. This justifies the choice of this idealized objective function to study the characteristics of synaptic connection within the preBötC network. The obtained network characteristics well may reflect the characteristics of actual network structure within the preBötC.

**Objective-CN2:** Initially, attempts were made to evolve composite networks towards Objectives-CN1 and CN3 alone. However, during the evolution process, the evolving networks illustrated a tendency to minimize Cost3, given by Eq (3), by reducing its inter-burst interval. Since the dynamics of neurons are such that they exhibit a period of quiescence soon after bursting, a shorter inter-burst interval ensures that neurons exhibit less sporadic bursting. Consequently leading to lower Cost3. Objective-2 was devised in order to suppress this phenomena. Addition of Objective-2 ensures that the evolutionary networks evolve to minimize Cost3 solely by rearranging the network synapses.

**Objective-CN3:** The relationship between intrinsically bursting and rest of the neurons within the ‘excitatory network’ may be analogous to that of ‘leader-follower relation’ found in social communities, with the former set of neurons acting as leaders and the later set of neurons as followers. Objective-1 was devised with the idea that for robust rhythm generation, it is desirable that this leader-follower relation between intrinsically bursting and rest of the neurons in the excitatory network is strong. Extending the sociological analogy further, it was presumed that inhibitory neurons act analogous to (constructive) social critiques—they criticise (inhibit) those activity of individual neurons which tend to disrupt the desirable harmony of ‘leader-follower relation’ between intrinsically bursting and rest of the neurons in the excitatory network. It is this idea that motivated us to formulate Objective-3.

**Scaling factors for Costs:** The scaling factors—50 for Cost1 [Eq (1)], 1.0 for Cost2 [Eq (2)] and 100 for Cost3 [Eq (3)]—were fixed by trial and error. Motivation was to evolve the networks towards the defined objectives with following priority order: Objective-CN2 (top priority), Objective-CN1 (second priority) and Objective-CN3 (third priority). The chosen scaling factors ensured that evolving networks evolved with their IBI tightly constrained close to desired value of 7s. This ensured that evolving networks primarily differed from each other in terms of their *non-trivial costs*—Cost1 (measuring network synchronicity) and Cost3 (measuring sporadic bursting of excitatory neurons); Cost2 (measuring the deviation of network’s IBI from the set value of 7s) is considered trivial cost here. This may be noted from reduction history of costs depicted in Fig 11 and S14 Fig.

**Objectives EN1 and EN2:** Objectives EN1 and EN2 used for evolving random excitatory networks are equivalent forms of objectives CN1 and CN2; simplified to a form so that they are applicable to excitatory networks. Since excitatory networks do not comprise any inhibitory neurons, no equivalent of Objective CN3 is used for evolving excitatory networks.

### 4.2 The structure of evolved networks

**Evolved excitatory network:** The common feature of the evolved network is to have ‘collector’ and ‘spreader’ neurons within network. Interestingly, neurons rich with out-going synapses are mostly pacemaker neurons in the core region while less readily excitable non-pacemaker cells in the activated region are rich in in-coming synapses. That is, the evolved network is rich with synapses having pacemaker and non-pacemaker neurons at pre- and post-synaptic ends, respectively.

The skewed/heavy tailed degree distribution of incoming synapses in the evolved network may be related to inhomogeneous cellular properties. Neurons in the ‘activated region’ require increasingly high degree of EPSPs provided by incoming synapses to make them exhibit synchronized bursting with rest of the network neurons, which results in the elongated tail towards the right in Fig 8C.

Our results suggest that random networks show better performance than SW networks in terms of synchronization (Fig 9). In conventional description of SW networks, all nodes within a graph are identical; there is no intrinsic difference between any two nodes. However, in the present study, *model* neurons act as nodes, which have varied intrinsic properties depending on the *g*_*Leak*_ and *g*_*NaP*_ values that are randomly allocated to them. Consequently, network burst synchronization depends on both network topology and intrinsic property of neurons at specific nodal positions. In a random network, a few intrinsically bursting neuron may *by chance* end up occupying nodal positions in network with above average out-going synapses. Such neurons end up making greater contribution in synchronizing the network by providing EPSPs to greater number of network neurons. However, a SW network is constrained by its characteristic that all nodes necessarily have (almost) identical number of neighbours; equivalently, equal number of outgoing synapses. This constraint limits the extent to which individual neurons may contribute towards network synchronization. It is probably this difference that renders SW network less effective at synchronizing the network when compared to the random network in Fig 9. Moreover, the same characteristic of SW network, i.e. all nodes have almost equal number of outgoing/incomming synapses, possibly limits the optimum network synchronization that may be achieved by a SW network. This may be the reason why the best evolved SW network using MM4 is inferior to the best evolved network using MM3 in Fig 10.

**Evolved composite network:** As in case of ‘evolved excitatory network’, it was common to find several ‘collector’ and ‘spreader’ neurons, of both excitatory and inhibitory types, within evolved composite networks. The chief features of evolved composite network is concisely summarized in Fig 15B, studying which following remarks may be made:

1. Similar to ‘evolved excitatory network’, evolved composite network also possesses rich projections from excitatory neurons in the core region to excitatory non-pacemaker neurons in the activated region. Excitatory neurons in the core region also project synapses to inhibitory neurons belonging to both core and activated regions. Thus, in essence, excitatory neurons in the core region may be thought of as a subpopulation of evolved composite network dictating the network’s rhythmic synchronous bursts through its phasic excitatory drive.
2. Inhibitory ‘core region’ neurons provide synaptic projection to excitatory ‘tonic region’ and ‘core region’ neurons. With the objectives employed for evolving the network, this may be rationalized as follows: (1) inhibitory synaptic inputs provided to excitatory tonic neurons help to minimize sporadic bursts of excitatory network, and (2) inhibitory synaptic inputs provided to excitatory ‘core region’ neurons most probably assists the excitatory network to exhibit network bursts with (desired) inter-burst interval of 7s; in the absence of this inhibitory inputs, the inter-burst interval of network is lower (see section 3.2). It is interesting to note that inhibitory neurons have little influence on excitatory ‘activated region’ neurons, primarily because unlike ‘tonic region’ and ‘core region’ neurons they do not participate in sporadic bursting.

As is discussed in section 4.1, the three objectives used for network evolution are (hypothesized) ‘idealizations’ of functional purpose of excitatory and inhibitory networks within the preBötC. Consequently, the connectivity characteristics of evolved composite network may be thought to reflect characteristics of an ‘idealization’ of preBötC network, referred to as ‘idealized network’ henceforth.

### 4.3 Comparisons with network structures previously proposed

Rekling et al. [26], based on dual recordings, have reported that synaptic connectivity among rhythmogenic (type-1) neurons within preBötC is ≈13%. The network structure within the preBötC was experimentally analyzed by Hartelt et al. [18]. Using a novel *in situ* imaging technique, they have shown that the network within the preBötC has a modular structure consisting of clusters having transverse (dorso-ventral) structure. They found that the mean number of cells within a cluster are 6.7±0.6 with each cell having 4.4±0.5 connections to other cells. For cells outside the clusters, the number of connections was considerably sparse, 2.1±0.4. Further, Carroll and Ramirez [3], based on cross-correlation analysis of multicellular experiments conducted on preBötC suggests that cellular connectivity probability is only about 1%. Based on these discrepant information, the choice of *SynFrac* ≈ 0.10–0.12 that we employed for evolving the excitatory and composite networks is probably a reasonable one. Recently Wang et al. [27], through simulation study found that for random networks, *SynFrac* ≈ 0.13 generates respiratory-like cycle periods. However, they used Rubin-Hayes preBötC neuron model [10], which is different from the neuron model that we have used in our study, and the network size is >200 neurons (nodes).

Gaiteri and Rubin [28] compared the performance of the connectivity topology within preBötC as observed by Hartelt et al. [18] (modular structure consisting of clusters) in terms of network burst synchronization with those of small-world, scale-free, random, and regularly structured networks. They found that, despite the functional role of synchronized bursting within the preBötC, synchronized bursting is generally weakest in the Hartelt network topology. However, in the work of Hartelt et al., clustering of neurons was largely based on their physical proximity rather than synaptic connectivity (see Figs 4 and 5 in Hartelt et al. [18]). Moreover, since the nature of cells (excitatory or inhibitory) is unknown, functionality of synapse (excitation or inhibition) is not taken into consideration in their study. Therefore, the Hartelt network topology may not represent the functional preBötC network structure. On the other hand, since our theoretical analysis of connectivity graphs does not consider the neuroanatomical structure of the preBötC, the ‘idealized network’ may not reflect the *real* network whose topology is bound to the neuroanatomical structure [29].

Gaiteri and Rubin [28] tested several combinations of network topology and cellular properties by positioning cells with specific cellular properties hierarchically. They found that random topology networks generated greatest global synchrony when bursting cells were located at central network positions, whereas placement of tonic firing cells at the most central locations was most effective in promoting network bursts in scale-free networks. Our study complements the findings of Gaiteri and Rubin by providing additional new results. In particular, our study suggests SW networks are *no better* than random networks as far as synchronization of neuronal network is concerned. The evolved excitatory network, which exhibits highest degree of network synchronization, possesses skewed/heavy-tailed degree distribution with rich synaptic projections extending from pacemaker neurons to non-pacemaker neurons. Further, our work shows that evolved ‘composite networks’ also have ‘spreader’ neurons in both excitatory and inhibitory sub-populations, whose cellular properties are in the ‘core region’.

Carroll and Ramirez [3] investigated the cycle-to-cycle variability of preBötC network activity, and concluded that respiratory neurons were stochastically activated with each burst, and intrinsically bursting pacemakers led some population bursts and followed others. They suggested that the variability could only be reproduced by sparsely connected network models at as less as 1 percent connectivity. However, they used random network topology consisting of only excitatory neurons. It is very likely that if the presence of inhibitory neurons are incorporated in the simulated network then higher burst onset variability may be achieved with higher fraction of all-to-all synaptic connections. Indeed, inter-burst interval becomes variable with inhibitory connections; one of the 12 evolved composite networks exhibited variable IBI (though the mean IBI remained ≈7s, as is set in objective-CN2; data not shown).

### 4.4 Uncertainty of cellular mechanisms of respiratory rhythm generation

In the present study, we used a mathematical model described by Purvis et al. [4] (which is modified version of model 1 in Butera et al. [23]) to formulate the dynamics of both excitatory and inhibitory neurons. However, there is uncertainty of the cellular mechanisms of respiratory rhythm generation. The models of the isolated preBötC network based on populations of neurons with INaP-dependent bursting properties could explain a number of experimentally observed features of preBötC neuron and network rhythmic behavior. Moreover, experimental studies confirmed that INaP is ubiquitous in preBötC neurons and endows both individual neurons and the network as a whole with intrinsic oscillatory bursting properties. However, the necessity of INaP for preBötC rhythm generation has been questioned, and still on debate [2, 24, 30]. A ‘group pacemaker’ model of preBötC bursting activity based on Ca^2+^-activated non-selective cationic current (ICAN), motivated and supported by experimental studies [31, 32], was proposed by Rubin et al. [10], in which Ca^2+^ accumulation by the activation of ionotropic and metabotropic glutamate receptors leads to the activation of ICAN, resulting in shaping the driving potential of the burst phase of neuronal and network activity. More recently, models of rhythmogenesis in preBötC combining mechanisms associated with INaP and ICAN [12, 13] have proposed. Although they still have all-to-all connections, they can reproduce variety of experimental results. The use of these mathematical models could produce different results, and further study is required.

### 4.5 Remarks on Simulated Annealing methodology

The fact that the random hill climbing algorithm, using MM1 or MM2 as mutation method, is intrinsically ‘constrained’ is already explained in section 2.3. While network initialization, *SynStrength* and *SynFrac* is fixed a priori. Once set, these values remain fixed during the entire evolutionary process (when network is evolved using random hill climbing algorithm). Consequently, the best evolved network achievable through evolutionary process is a function of these initially fixed parameters. The product (*SynStrength* × *SynFrac*), is one of the most important parameter that is required to be set correctly during network initialization so as to ensure its proper evolution. This is relatively straight forward to comprehend for the case of evolution of ‘excitatory network’ towards complete synchronicity (section 3.1): when the initialized value of product (*SynStrength* × *SynFrac*) is low, the network fails to evolve completely to exhibit full synchronization; a fraction of neurons belonging to the ‘activated region’ remain silent and fail to participate in population burst. On the other hand, when the initialized value of product (*SynStrength* × *SynFrac*) is high, the network does evolve to exhibit full synchronization but possess several redundant synapses in it. Both cases hinder identification of essential features of network connectivity.

However, in the simulated annealing algorithm used for the present study, by allowing *SynFrac* to vary, we let the value of product *SynStrength* × *SynFrac* evolve along with the evolving network. This extra degree of freedom introduced in the simulated annealing algorithm, allows the network to evolve to full synchronization even when initialized value of product *SynStrength* × *SynFrac* is low or high. Moreover, since the simulated annealing algorithm preferentially selects networks with lower synapse count in every iteration of evolution, the redundant synapse count in the evolved network is minimal. These features of the simulated annealing algorithm are advantageous when it is used to evolve ‘composite networks’.

Another appealing aspect of simulated annealing algorithm is that it is more prolific in producing favorable network mutations when compared with random hill climbing. Random hill climbing algorithm utilizes mutation methods which preserve the total synapse count. Consequently, for a favorable network mutation to occur two operations—the random deletion of synapses *and* random addition of equal number of synapses—must simultaneously combine in a ‘positive way’. The frequency of occurrence of such a favorable mutation is substantial when the network is composed of only excitatory neurons. However, when the network is composed of both excitatory and inhibitory neurons, the frequency of occurrence of such a favorable mutation decreases considerably. To a lesser degree, the increased size of composite network reduces the probability of finding favorable mutations as well. In contrast to this, success of simulated annealing algorithm in producing a favorable network mutation depends only on one operation—addition (*or* deletion) of synapses—and hence it produces favorable network mutations with greater frequency.

It is basically for the reasons specified in preceding two paragraphs that we directly employed simulated annealing algorithm for evolving the composite network. Note however that MM2, which is a total synapse preserving mutation method, is ‘active’ in parallel (see Fig 11) all the while when simulated annealing algorithm is implemented.

Scale-free network, which has extreme skewed/heavy-tailed degree distribution, emerges when nodes and connections are added with a higher probability to connect with a highly connected cells [21] or when connections are removed with a probability that is inversely related to the sum of their first- and second- order connectivity [33]. In the present study, we demonstrated that skewed/heavy-tailed degree distribution within network may emerge even when nodes (neurons) and connections (synapses) are added or eliminated based *not* on the topology of the network but on the behavior of the network, i.e., the synchronicity of the network activity (note: the differential influence of individual neurons (nodes) on network behavior on account of their differing *intrinsic characteristic* is essential here). Such addition and elimination of nodes and connections—simulated annealing—may be viewed as *partially* analogous to addition and elimination of neurons and synapses associated with the development of brain structures during ontogeny [34]. Simulating ontogenical evolution of network would require updating of the intrinsic property (*g*_*Leak*_/*g*_*NaP*_ values) of constituting neurons as well, which is not incorporated in the present work.

## 5 Conclusion

In summary, we conclude the following:

1. An effective evolutionary algorithm is developed to evolve the synaptic connectivity within a neuronal network towards desired objective function. The developed algorithm is sufficiently generic—it can handle neuronal networks comprising both excitatory and inhibitory neurons.
2. Evolved excitatory network, which is an ‘idealization’ of excitatory subnetwork of preBötC, possesses the following features:
  - skewed/heavy-tailed degree distribution among network neurons. Network is rich with synapses projecting from tonic/core region neurons to non-pacemaking neurons in the activated region.
  - superior network synchronization when compared to a small-world network with identical number of synapses.
3. Evolved composite network, which is an ‘idealization’ of preBötC network, possesses following features:
  - excitatory subnetwork retains the overall salient features of evolved excitatory network mentioned above.
  - excitatory ‘core region’ neurons dictate the network’s rhythmic synchronous bursts through its phasic excitatory drive.
  - inhibitory subnetwork influences excitatory subnetwork primarily through ‘core region’ neurons; their inhibitory synaptic inputs (1) minimize sporadic bursting of excitatory tonic neurons, and (2) assists excitatory ‘core region’ neurons to exhibit network bursts with desirable inter-burst interval.

## Appendix A Network Mutation methods

Four different network mutation methods were devised.

1. **Mutation Method-1 (MM1):** Network mutation is exercised by deleting a set of synapses followed by adding an equivalent number of synapses to the network. To determine synapses to be deleted, two neurons, say *n*_1_ and *n*_2_, are randomly chosen from the neuronal group and then, their respective neighborhoods *N*_1_ and *N*_2_ were defined. To define neighborhoods *N*_1_ and *N*_2_, we define an Euclidean metric
  $$\begin{array}{c} D_{ij}=\sqrt{{\left(g_{Leak_{i}}-g_{Leak_{j}}\right)}^{2}+{\left(g_{NaP_{i}}-g_{NaP_{j}}\right)}^{2}} \end{array}$$
  over *g*_*Leak*_/*g*_*NaP*_-parametric space depicted in S1 Fig, enabling us to define distance between two arbitrary neurons *n*_*i*_ and *n*_*j*_. Consequently, neighborhood *N*_1_ is composed of all neurons *n*_*i*_ ≡ (*g*_*Leak*_*i*__, *g*_*NaP*_*i*__) corresponding to $D_{1i}=\sqrt{{\left(g_{Leak_{i}}-g_{Leak_{n1}}\right)}^{2}+{\left(g_{NaP_{i}}-g_{NaP_{n1}}\right)}^{2}}<\delta \left(N_{1}\right)$, where *δ*(*N*_1_) is the radius of neighborhood *N*_1_ with neuron *n*_1_ ≡ (*g*_*Leak*_1__, *g*_*NaP*_1__) at its center; *δ*(*N*_1_) is randomly specified during each mutation process. Neighborhood *N*_2_ is similarly computed. A random fraction of synapses directed from *N*_1_ to *N*_2_ is deleted during each mutation step. An equal number of synapses directed from neighborhood *N*_3_ to neighborhood *N*_4_, is subsequently added to the network; where *N*_3_ and *N*_4_ are neighborhoods of two additional randomly selecting neurons *n*_3_ and *n*_4_ from the neuronal network.
  MM1 is devised on the assumption that neurons with similar values of *g*_*Leak*_ and *g*_*NaP*_, and thus occupying same local neighborhood on *g*_*Leak*_/*g*_*NaP*_-parametric space depicted in S1 Fig, serve similar purpose within the neuronal network. Consequently, synapses directed from neurons within one local neighborhood to neurons within another local neighborhood of *g*_*Leak*_/*g*_*NaP*_-parametric space also serve similar purpose within the neuronal network, and thus may be added or deleted together. This method of mutating the network ensures that all the added/deleted synapses effect favorable or unfavorable change to the network unanimously (more or less) and thus is more effective than simply randomly adding or deleting a fraction of network’s total synapse.
  Additional remark: while employing MM1 to mutate neuronal network composed of both excitatory and inhibitory network (composite network), caution is exercised such that all neurons within neighbourhoods *N*_1_, *N*_2_, *N*_3_ and *N*_4_ are of the same kind (all excitatory or all inhibitory).
2. **Mutation Method-2 (MM2):** This method is comprised of two distinct mutations depicted in S2 Fig. In particular, two different neurons *n*_1_ and *n*_2_ are chosen at random and a fraction of incoming (or outgoing) synapses of *n*_1_ are rewired to *n*_2_. These changes are effected by appropriately changing the elements of connectivity matrix (Fig 3). For every iteration of network evolution, one of these two mutations is randomly selected and used for network mutation.
  Note that intrinsic to MM1 is the tacit assumption that neurons with similar values of *g*_*Leak*_ and *g*_*NaP*_ serve similar purpose within the neuronal network. MM2 is free from any such assumptions, and hence is more general than MM1.
  While employing MM2 to mutate neuronal network composed of both excitatory and inhibitory network, caution is exercised that all synapses to be rewired are of the same kind (all excitatory or all inhibitory). Note that here it is possible that excitatory synapses from E-E part of the adjacency matrix may get reallocated to E-I part (Fig 3).
3. **Mutation Method-3 (MM3):** This mutation method has following two subtypes:
  **MM3(SynAdd)** This adds new synapses to the network. *Method:* A neuron in the network is randomly chosen and a small fraction of (maximum possible) incoming or outgoing synapses are added to it. S3A and S3B Fig together constitute MM3(SynAdd).
  **MM3(SynDel)** This deletes existing synapses from the network. *Method:* A neuron in the network is randomly chosen and a small fraction of all incoming or outgoing synapses to it are deleted. S3C and S3D Fig together constitute MM3(SynDel).
  Both subtypes may be implemented in conjunction (i.e. some new networks may be obtained by employing MM3(SynAdd) and others by using MM3(SynDel)) or in isolation. To prevent sudden large change in network topology in one mutation step, maximum number of synapses that may be added by MM3(SynAdd) or deleted by MM3(SynDel), during mutation is restricted to 5 synapses.
  While employing MM3 to mutate neuronal network composed of both excitatory and inhibitory network, caution is exercised that all synapses added or deleted from the network are of same kind (all excitatory or all inhibitory).
4. **Mutation Method-4 (MM4):** S4 Fig describes MM4. Though, S4 Fig illustrates MM4 by interchanging the position of a pair of neurons at a time, in actual implementation of MM4 positions of 2–5 randomly chosen neurons are interchanged at a time. It may be noted that MM4 is fundamentally different than the above mutation methods; unlike above mutation methods, MM4 preserves the structure of network connectivity, consequently initial and mutated networks remain *homeomorphic* to each other.

Except for MM3, all other three mutation methods described above, preserve the total number of synaptic connections within the neuronal group. All three methods simply keep rearranging the synaptic connections within neuronal network so as to help it exhibit desired population behavior.

It is remarked that all four mutation methods (MM1-4) are custom-made algorithm, devised for the sole purpose of serving the application at hand (i.e. to evolve the neuronal network connectivity). However, the key ideas employed in these mutation methods, particularly MM2-4, are similar to those found in ‘swap mutation’ and ‘scramble mutation’ of Genetic Algorithm [35].

## Appendix B Computation of Cost3

The procedure for computing ‘total sporadic burst count’ and ‘total sporadic burst count possible’ required for determination of Cost3 using Eq (3) is illustrated in S9 Fig. In general, for a given neuron, ‘total sporadic burst count’ is the sum-total of all time-bins capturing its spiking/bursting activities, which are not synchronous with network’s population burst. For example, referring to S9 Fig, for the inter-burst interval depicted, neuron-1’s sporadic burst count is 3+4 = 7.

Since, pre-inspiratory (pre-I) bursting of a subset of network neurons is an essential precursor to help the entire network exhibit a synchronized population burst subsequently, short pre-I activity of neurons—for example, pre-I bursting of neurons-3 and 4 in S9 Fig—are not considered as sporadic bursts. However, in case of neurons exhibiting elongated pre-I activity—for example, neuron-5 and (tonic) neuron-7 in S9 Fig—pre-I activity only up to a specified pre-I time-bin is discounted as non-sporadic bursting. In S9 Fig, for clarity of depiction, pre-I activity up to 5 time-bins are discounted as non-sporadic activity; in actual implementation [ie. for computing Cost3 using Eq (3)] pre-I activity up to 7 time-bins were discounted as non-sporadic activity.

Similarly, short post-I activity of neurons—for example, post-I bursting of neurons-2 and 4 in S9 Fig—are not considered as sporadic bursts. In case of neurons exhibiting elongated post-I activity—for example, neuron-6 and (tonic) neuron-7 in S9 Fig—post-I activity only up to a specified post-I time-bin is discounted as non-sporadic bursting. In S9 Fig, for clarity of depiction, post-I activity up to 5 time-bins are discounted as non-sporadic activity; in actual implementation post-I activity up to 7 time-bins were discounted as non-sporadic activity.

Taking the above conventions into account, ‘total sporadic burst count possible’ for a given neuron is the sum-total of all the time-bins during which neuron’s activity would potentially be considered as sporadic bursting. S9 Fig provides a summary to help clarify the conventions employed.

## Appendix C Evolving a network employing Simulated Annealing Algorithm

Initially, several explorations were done to evolve the composite network using simulated annealing ‘manually’. Details of one such ‘manual’ attempt is provided in S3 Text. Based on the experience gained from these explorations and to remove user intervention on the network’s evolution history, a ‘rigid’ time-line for various mutation methods influencing network’s evolution was defined. All 12 composite networks were then subjected to this ‘pre-defined mutation time-line’ and allowed to evolve.

Referring to Fig 11, the course of ‘pre-defined mutation time-line’ may be divided into three different phases

**Iterations 0–1000:** All mutation methods—MM2, MM3(SynAdd) and MM3(SynDel)—are active. As the network continues to evolve, the Cost (Cost1+Cost2+Cost3) continues to diminish and reaches a stage where rate of decrease of Cost per iteration becomes relatively small. This initial phase of network evolution is accompanied with increase in synapse count in the network.
**Iterations 1000–3400:** Mutation methods MM3(SynDel) is periodically deactivated and activated for a period of 100 and 200 iterations, respectively. During the period when MM3(SynDel) is inactive, networks evolves under the influence of mutation methods MM2 and MM3(SynAdd); network’s synapse count tends to increase. During the period when MM3(SynDel) is active, networks evolves under the influence of all three mutation methods—MM2, MM3(SynAdd) and MM3(SynDel); this helps network ‘relax’. It also ensures that only Cost-minimizing synapses are added to the network by effecting deletion of redundant synapses present in the network.
**Iterations 3400–6400:** Mutation methods MM3(SynAdd) is periodically deactivated and activated for a period of 100 and 200 iterations, respectively. During the period when MM3(SynAdd) is inactive, networks evolves under the influence of mutation methods MM2 and MM3(SynDel); network’s synapse count tends to decrease.

The evolved composite network obtained at the end of this iterative random mutation and selection process is expected to have captured essential features of desirable network connectivity pattern.

Note that the utilized evolutionary process comprises of only one cycle of increase and decrease in network’s synapse count. Conventional paradigm of simulated annealing algorithm suggests execution of more than one such cycle. However, since care has been exercised to periodically ‘relax’ the network during its evolution process so that the effect of random drift is minimal, one cycle was considered sufficient. The experience gained from preliminary explorations described in section S3 Text does corroborates this.

## Supporting Information

S1 Fig Mutation method-1. Four different neighborhoods *N*_1_, *N*_2_, *N*_3_ and *N*_4_ are randomly selected on neuron property plane. A fraction of synaptic connections directed from *N*_1_ to *N*_2_ is deleted, and an equal number of synaptic connections directed from *N*_3_ to *N*_4_ is added.
(PDF)

S2 Fig Mutation method-2. Two different neurons *n*_1_ and *n*_2_ are chosen at random. Then (A) a fraction (value of this fraction is randomly chosen at each step) of *incoming* synapse to neuron *n*_1_ (indicated in gray color) are removed and allocated to neuron *n*_2_, and (B) a fraction of *outgoing* synapse from neuron *n*_1_ are removed and allocated to neuron *n*_2_. These changes are effected by appropriately changing the elements of connectivity matrix.
(PDF)

S3 Fig Mutation method-3. A neuron within the neuronal group is randomly selected, then (A) a *small* fraction (value of this fraction is randomly chosen at each step) of ‘maximum possible *incoming* synapse that can be added to the selected neuron’ is added (indicated in gray color); in the case presented, ‘maximum possible’ incoming synapse that may be added to selected neuron is 3, (B) a *small* fraction of ‘maximum possible *outgoing* synapse that can be added to the selected neuron’ is added, (C) a *small* fraction of all *incoming* synapse to the selected neuron is deleted, and (D) a *small* fraction of all *outgoing* synapse from the selected neuron is deleted. (A) and (B) together constitute MM3(SynAdd), while (C) and (D) together constitute MM3(SynDel); see Appendix A.
(PDF)

S4 Fig Mutation method-4. Two different neurons *n*_1_ and *n*_2_ are chosen at random and their position within the network is interchanged. This change is effected by exchanging the rows and columns of connectivity matrix corresponding to neurons *n*_1_ and *n*_2_, respectively.
(PDF)

S5 Fig Intrinsic interburt periods of network neurons. A & B: Intrinsic interburt periods of 80 neurons depicted in Fig 5A & 5C. Neurons depicted by big-encircled dots exhibit tonic activity at the set value of *g*_*tonic*_ (their interburst period is zero). Neurons depicted by hollow-tiny circles remain silent at the set value of *g*_*tonic*_ (they have no interburst period).
(PDF)

S6 Fig Histograms depicting distribution of inter-burst periods among various random networks. (Refer S1 Table for network A-E nomenclature.)
(PDF)

S7 Fig Depiction of evolution of ‘excitatory network’ in terms of ‘Cost’ minimization. Cost1 and Cost2 are given by Eqs (1) and (2), respectively. For network evolution, objective is to minimize (Cost1+Cost2). Numbers depicted along the curve represents ‘total number of iteration’ it took to evolve the network to that state beginning with initial random network. Inset show zoomed view of ‘Cost minimization’ during network evolution.
(PDF)

S8 Fig Evolution of cumulative curves (CC) depicting participation level of individual neurons to each population burst. CC_objective_: CC corresponding to the desired case when every neuron in the network bursts with every population burst. CC_initial_, CC_MM1_ and CC_MM3_ are CC corresponding to network activities depicted in Fig 6A ‘Initial’, ‘Evolved using MM1’ and ‘Further evolved using MM3’, respectively. CC_1_, CC_2_ and CC_3_ are representative intermediate CC obtained during the evolution process.
(PDF)

S9 Fig Cost3 computation. Illustration for computing ‘Total sporadic burst count’ and ‘Total sporadic burst count possible’ required for computing Cost3 using Eq (3). ‘Left’: Raster plot for seven hypothetical neurons exhibiting different kind of bursting activities (remarked in rightmost column of table on the ‘Right’) in the interval between two successive population bursts. Time bins marked with grey colored raster data are counted as sporadic bursts. ‘Right’: Table indicating ‘total sporadic burst possible’ and ‘total sporadic burst actually exhibited’ by each neurons between the inter-burst interval. Refer Appendix B for further explanation.
(PDF)

S10 Fig Testing the effectiveness of MM4. (A,B & C) Network activity of NET1, initially and after evolving it using MM4. Objective of evolution is to minimize (Cost1+Cost2) as defined by Eqs (1) and (2), respectively. (D) Evolution of cumulative curves (CC) depicting participation level of individual neurons to each population burst. CC_objective_: CC corresponding to the desired case when every neuron in the network bursts with every population burst. CC_MM4_ is CC corresponding to network activity depicted in (A)‘Evolved NET1 using MM4’. CC_0_, CC_1_, CC_2_ and CC_3_ are representative intermediate CC obtained during the evolution process. CC_MM3_ is CC corresponding to evolved excitatory network by method described in section 2.5 (it is the same CC_MM3_ curve as is depicted in S8 Fig).
(PDF)

S11 Fig Effect of removing IPSPs in the evolved composite network. Effect of elimination of IPSPs within the evolved composite network (same evolved network as depicted in Fig 12 ‘Evolved using Simulated annealing’). Elimination of IPSPs produced following changes: (1) Networks inter-burst interval (IBI) decreased from 7.1s to 6.64s, sporadic bursting of excitatory neurons (neurons 0–80) increased (quantitatively, value of Cost3 increased from 2.53 to 5.87), and (3) activity of inhibitory neurons are better synchronized with each population burst (apparent from visual inspection).
(PDF)

S12 Fig Histograms indicating distribution of neurons with various degrees of out-going and incoming synapses in the evolved composite network depicted in Fig 13. (PDF)

S13 Fig Evolved composite network for another evolutionary run. Synaptic distribution among neurons of evolved network for a different evolutionary run but with identical *SynStrength* and *SynFrac* values as in Fig 13. S14 Fig depicts the evolutionary history of this composite network. Initial synaptic distribution among neurons is randomly distributed with overall *SynFrac* = 0.06, which is lower than that of evolved network (*SynFrac* ≈ 0.10) and hence is not shown.
(PDF)

S14 Fig Evolutionary history of the evolved composite network depicted in S13 Fig. (A) Depiction of evolution of network in terms of ‘Cost’ minimization. Cost1, Cost2 and Cost3 are given by Eqs (1), (2) and (3), respectively. For network evolution, objective is to minimize (Cost1+Cost2+Cost3). Vertical color stripes indicates mutation methods that were employed during various stages for evolving the network. For further explanation see Appendix C. (B) Variation of synapses within the network during evolution process. Nomenclature: EEsyn, EIsyn, IEsyn and IIsyn are total synapses within EE, EI, IE and II part of adjacency matrix, see Fig 3C.
(PDF)

S15 Fig Evolutionary history of a *manually* evolved composite network. (A) Depiction of evolution of network in terms of ‘Cost’ minimization. Cost1, Cost2 and Cost3 are given by Eqs (1), (2) and (3), respectively. For network evolution, objective is to minimize (Cost1+Cost2+Cost3). Vertical color stripes indicates mutation methods that were employed during various stages for evolving the network. For further explanation see Appendix C. (B) Variation of synapses within the network during evolution process. Nomenclature: EEsyn, EIsyn, IEsyn and IIsyn are total synapses within EE, EI, IE and II part of adjacency matrix, see Fig 3C.
(PDF)

S1 Table Various network types and their relevance to ‘networks used for evolving’. (PDF)

S1 Text Intrinsic bursting frequency of constituting neurons and Network IBI. (PDF)

S2 Text Testing the effectiveness of MM4. (PDF)

S3 Text Manual explorations at evolving composite network employing Simulated Annealing Algorithm. (PDF)
