# Supplementary figures and images for: Emergent Network Topology within the Respiratory Rhythm-Generating Kernel Evolved In Silico
Source: PLoS One. 2016 May 6;11(5):e0154049. doi: 10.1371/journal.pone.0154049 (PMC4859517; doi:10.1371/journal.pone.0154049)

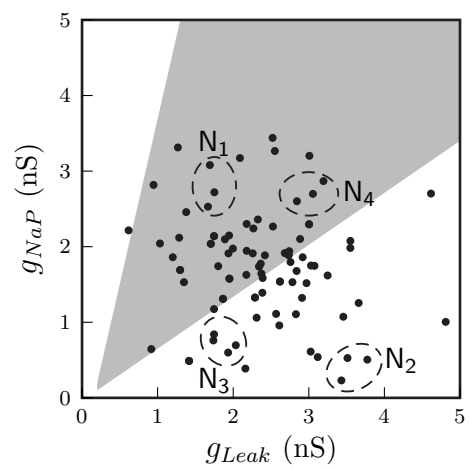

Supplement: S1 Fig — Four different neighborhoods N1, N2, N3 and N4 are randomly selected on neuron property plane. A fraction of synaptic connections directed from N1 to N2 is deleted, and an equal number of synaptic connections directed from N3 to N4 is added. (PDF) [file pone.0154049.s001.pdf]

Before Mutation

After Mutation

**A**

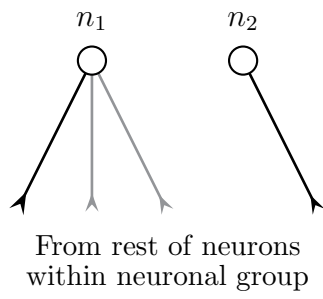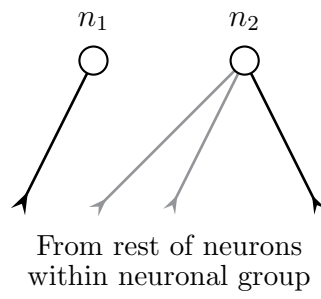

**B**

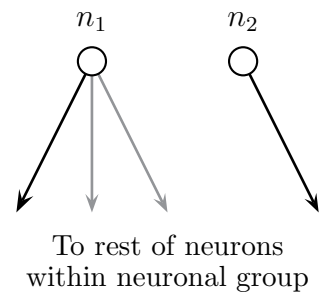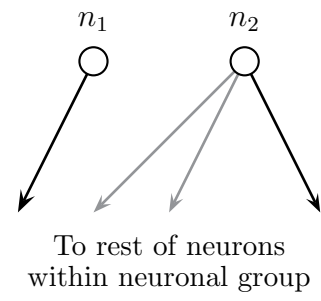

Supplement: S2 Fig — Two different neurons n1 and n2 are chosen at random. Then (A) a fraction (value of this fraction is randomly chosen at each step) of incoming synapse to neuron n1 (indicated in gray color) are removed and allocated to neuron n2, and (B) a fraction of outgoing synapse from neuron n1 are removed and allocated to neuron n2. These changes are effected by appropriately changing the elements of connectivity matrix. (PDF) [file pone.0154049.s002.pdf]

## Before Mutation

**A**

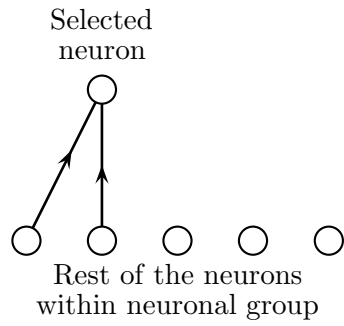

## After Mutation

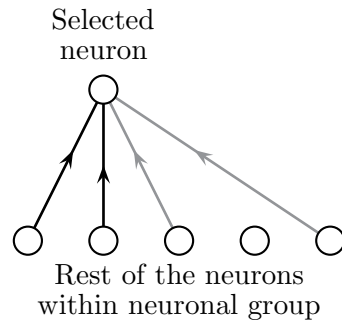

**B**

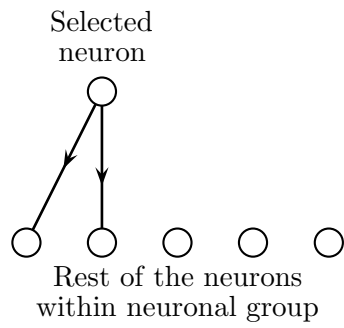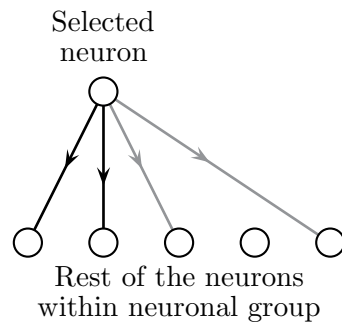

**C**

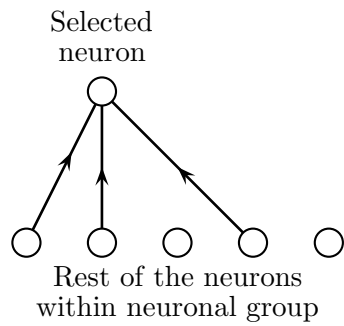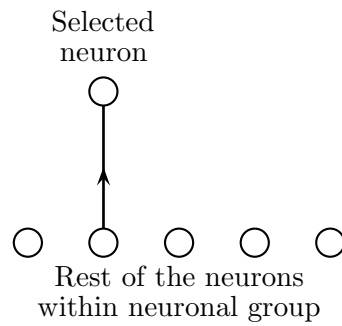

**D**

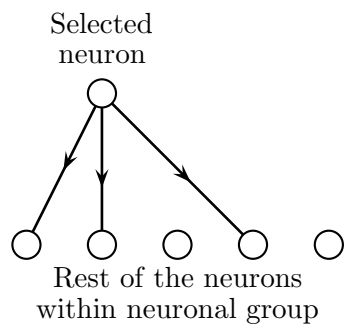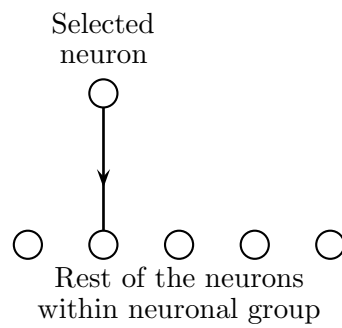

Supplement: S3 Fig — A neuron within the neuronal group is randomly selected, then (A) a small fraction (value of this fraction is randomly chosen at each step) of ‘maximum possible incoming synapse that can be added to the selected neuron’ is added (indicated in gray color); in the case presented, ‘maximum possible’ incoming synapse that may be added to selected neuron is 3, (B) a small fraction of ‘maximum possible outgoing synapse that can be added to the selected neuron’ is added, (C) a small fraction of all incoming synapse to the selected neuron is deleted, and (D) a small fraction of all outgoing synapse from the selected neuron is deleted. (A) and (B) together constitute MM3(SynAdd), while (C) and (D) together constitute MM3(SynDel); see Appendix A. (PDF) [file pone.0154049.s003.pdf]

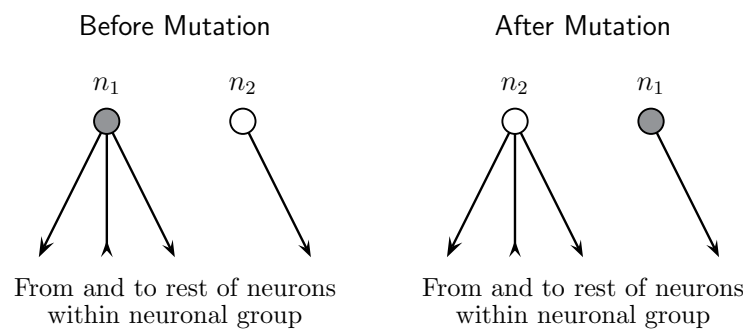

Supplement: S4 Fig — Two different neurons n1 and n2 are chosen at random and their position within the network is interchanged. This change is effected by exchanging the rows and columns of connectivity matrix corresponding to neurons n1 and n2, respectively. (PDF) [file pone.0154049.s004.pdf]

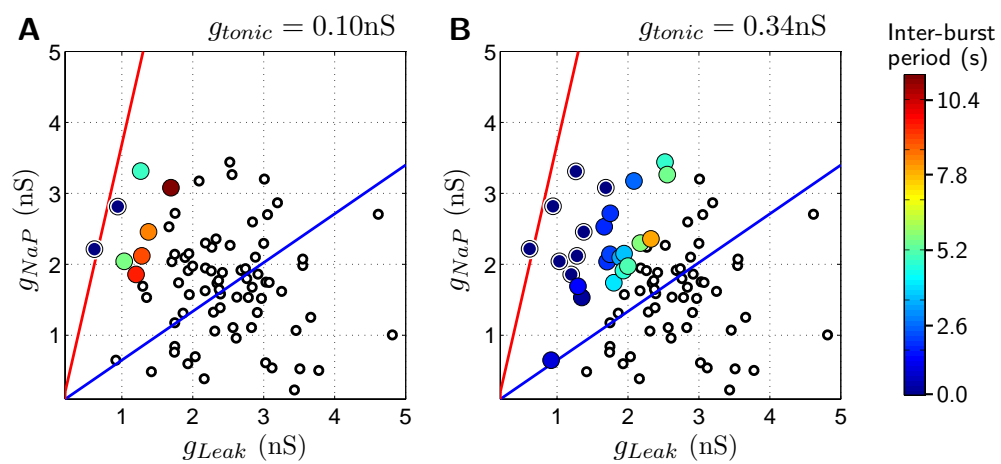

Supplement: S5 Fig — A & B: Intrinsic interburt periods of 80 neurons depicted in Fig 5A & 5C. Neurons depicted by big-encircled dots exhibit tonic activity at the set value of gtonic (their interburst period is zero). Neurons depicted by hollow-tiny circles remain silent at the set value of gtonic (they have no interburst period). (PDF) [file pone.0154049.s005.pdf]

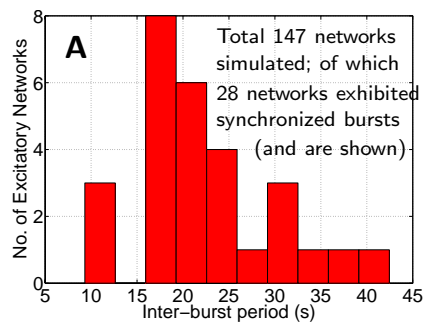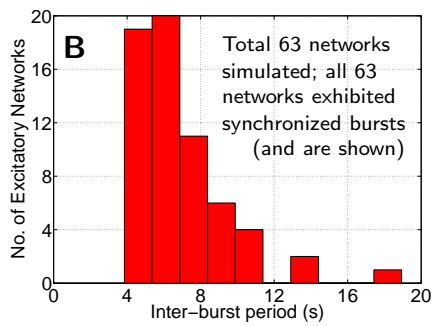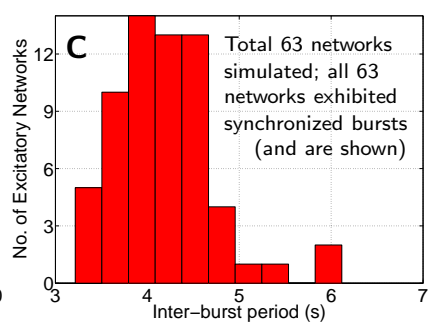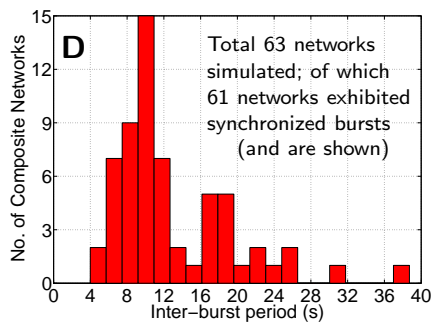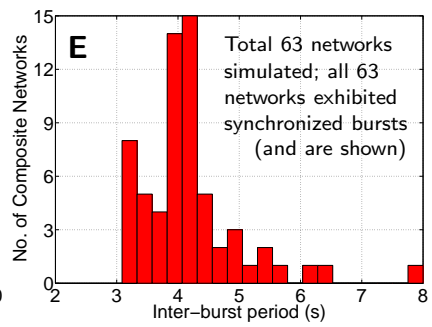

Supplement: S6 Fig — (Refer S1 Table for network A-E nomenclature.) (PDF) [file pone.0154049.s006.pdf]

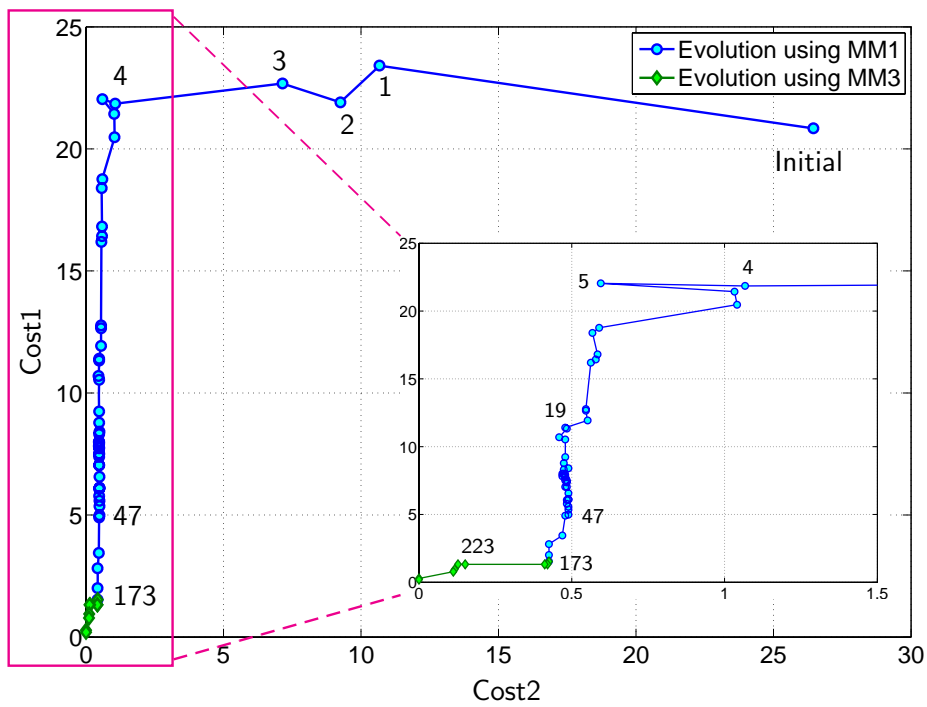

Supplement: S7 Fig — Cost1 and Cost2 are given by Eqs (1) and (2), respectively. For network evolution, objective is to minimize (Cost1+Cost2). Numbers depicted along the curve represents ‘total number of iteration’ it took to evolve the network to that state beginning with initial random network. Inset show zoomed view of ‘Cost minimization’ during network evolution. (PDF) [file pone.0154049.s007.pdf]

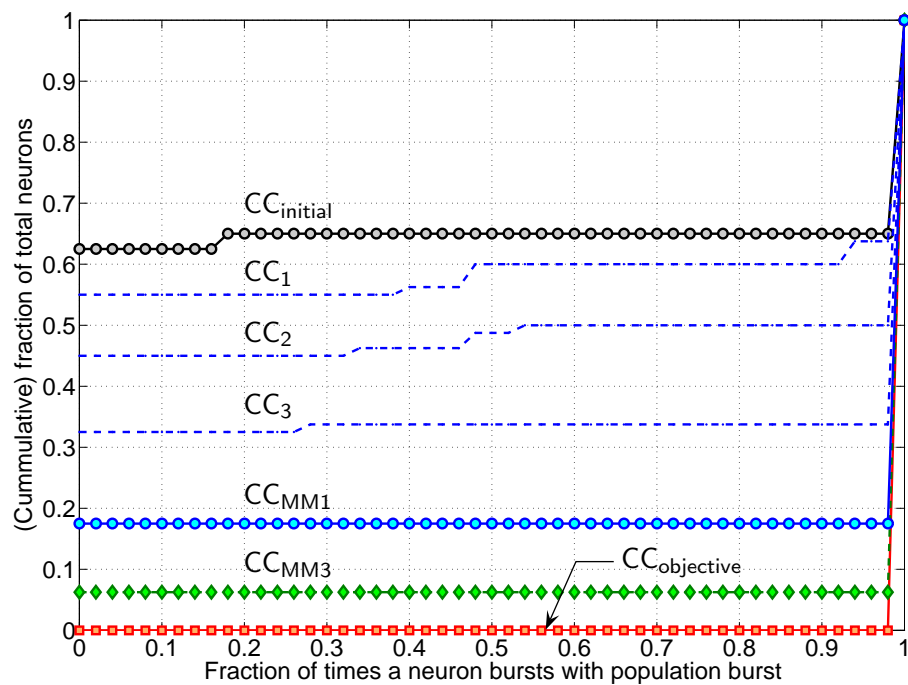

Supplement: S8 Fig — CCobjective: CC corresponding to the desired case when every neuron in the network bursts with every population burst. CCinitial, CCMM1 and CCMM3 are CC corresponding to network activities depicted in Fig 6A ‘Initial’, ‘Evolved using MM1’ and ‘Further evolved using MM3’, respectively. CC1, CC2 and CC3 are representative intermediate CC obtained during the evolution process. (PDF) [file pone.0154049.s008.pdf]

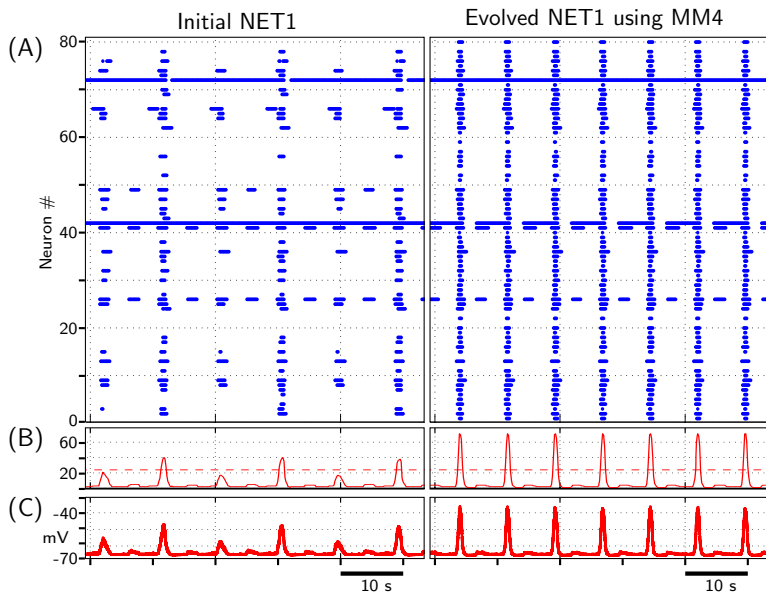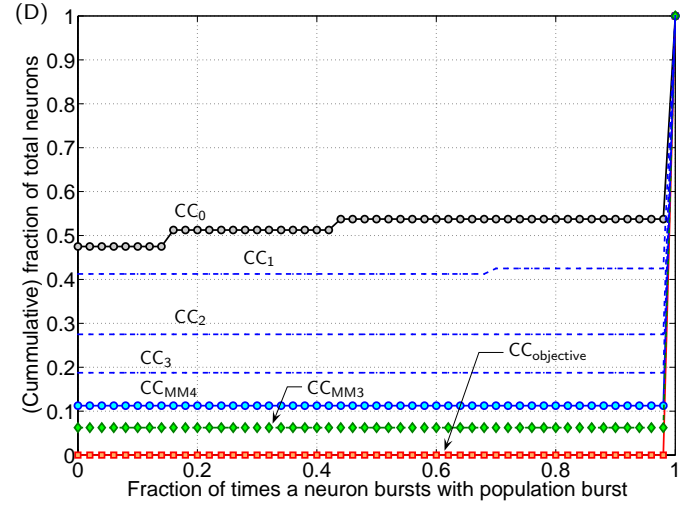

Supplement: S10 Fig — (A,B & C) Network activity of NET1, initially and after evolving it using MM4. Objective of evolution is to minimize (Cost1+Cost2) as defined by Eqs (1) and (2), respectively. (D) Evolution of cumulative curves (CC) depicting participation level of individual neurons to each population burst. CCobjective: CC corresponding to the desired case when every neuron in the network bursts with every population burst. CCMM4 is CC corresponding to network activity depicted in (A)‘Evolved NET1 using MM4’. CC0, CC1, CC2 and CC3 are representative intermediate CC obtained during the evolution process. CCMM3 is CC corresponding to evolved excitatory network by method described in section 2.5 (it is the same CCMM3 curve as is depicted in S8 Fig). (PDF) [file pone.0154049.s010.pdf]

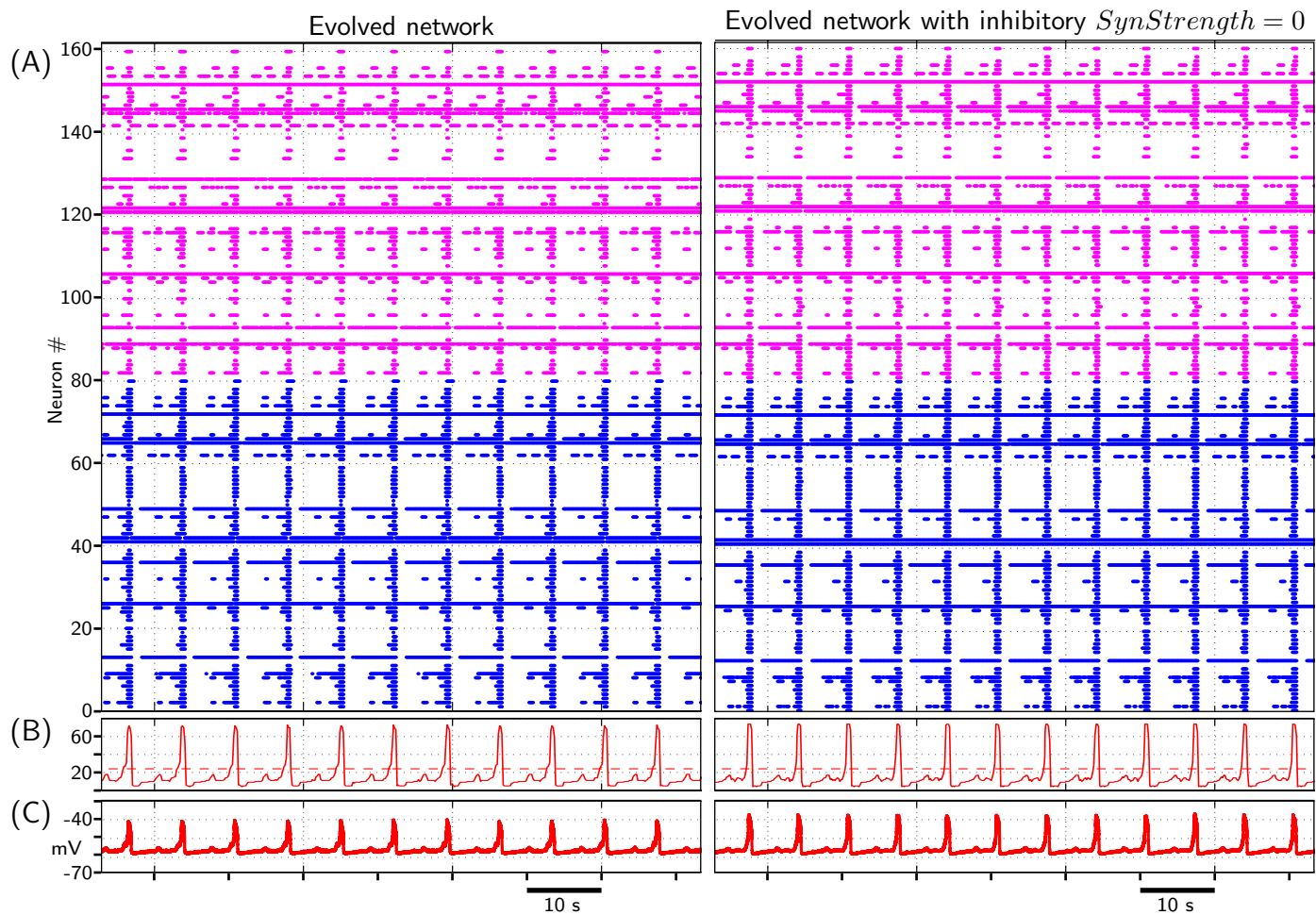

Supplement: S11 Fig — Effect of elimination of IPSPs within the evolved composite network (same evolved network as depicted in Fig 12 ‘Evolved using Simulated annealing’). Elimination of IPSPs produced following changes: (1) Networks inter-burst interval (IBI) decreased from 7.1s to 6.64s, sporadic bursting of excitatory neurons (neurons 0–80) increased (quantitatively, value of Cost3 increased from 2.53 to 5.87), and (3) activity of inhibitory neurons are better synchronized with each population burst (apparent from visual inspection). (PDF) [file pone.0154049.s011.pdf]

Out-going synapses  
(Final)

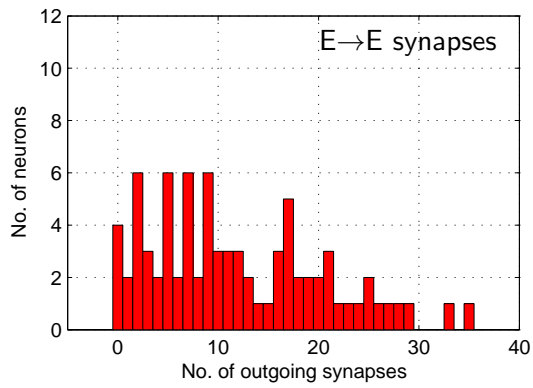

In-coming synapses  
(Final)

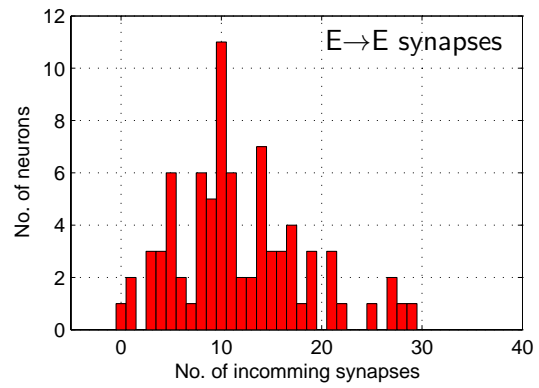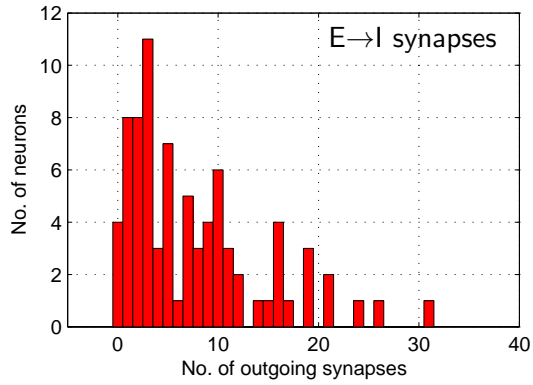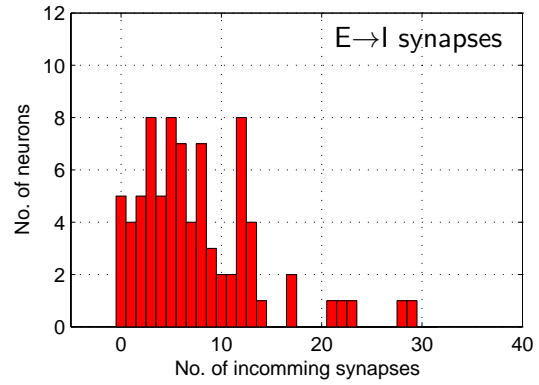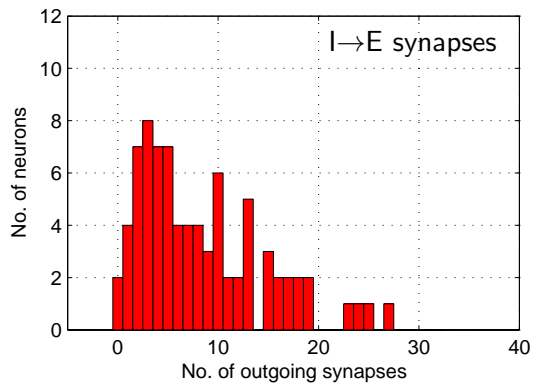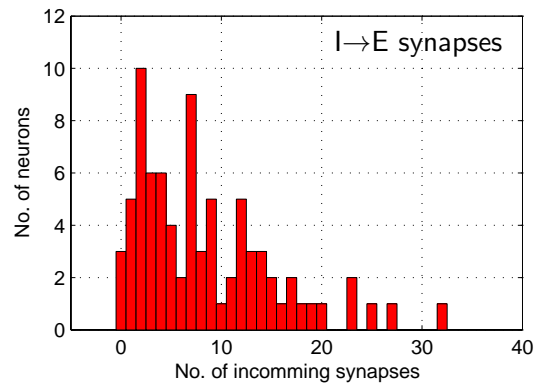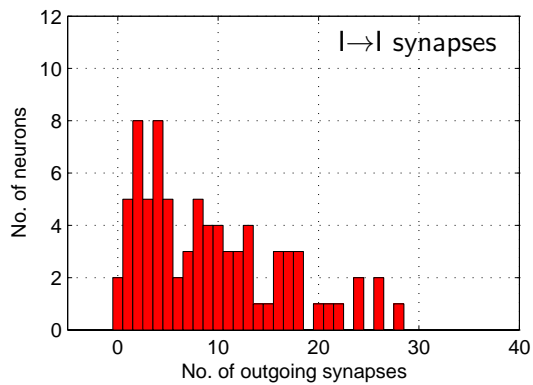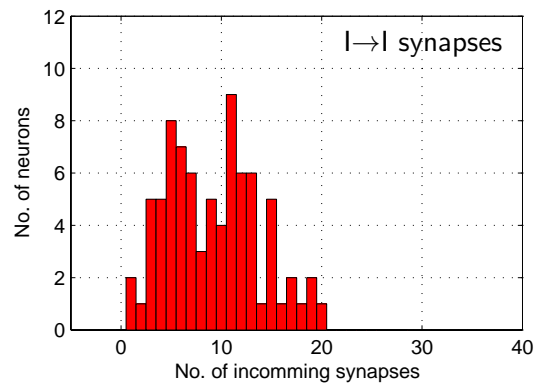

Supplement: S12 Fig — (PDF) [file pone.0154049.s012.pdf]

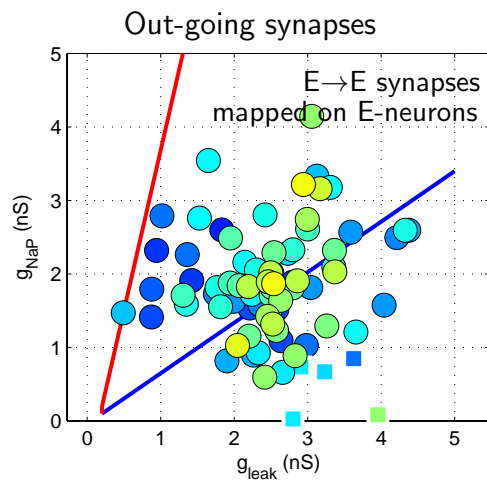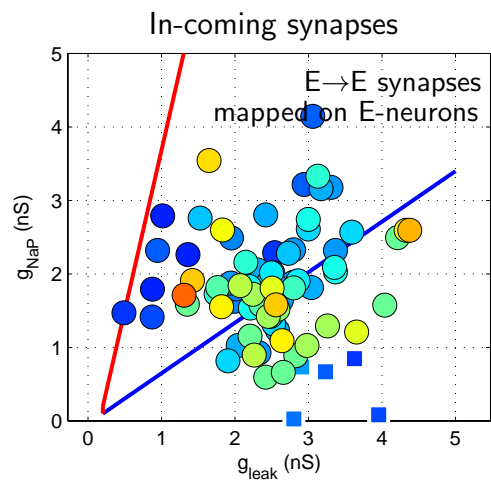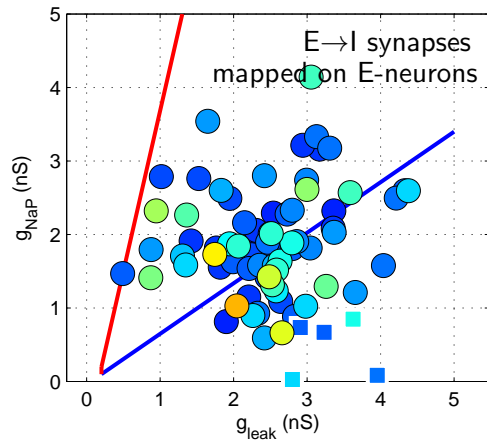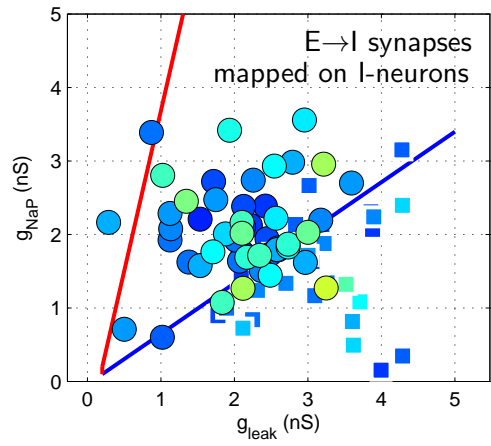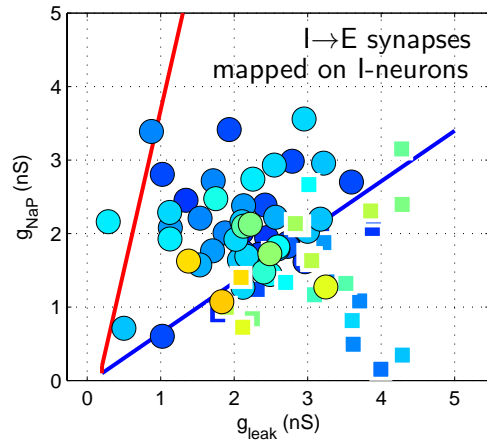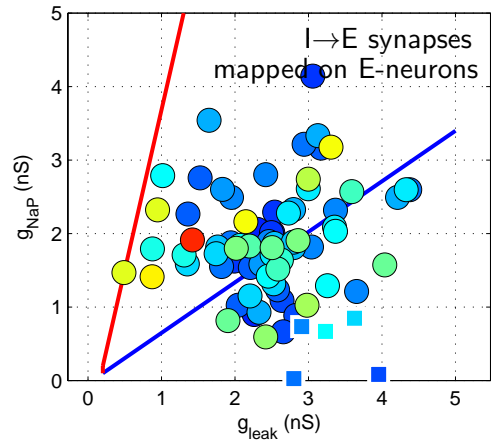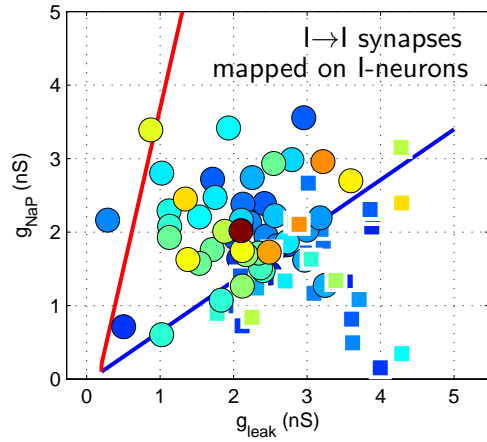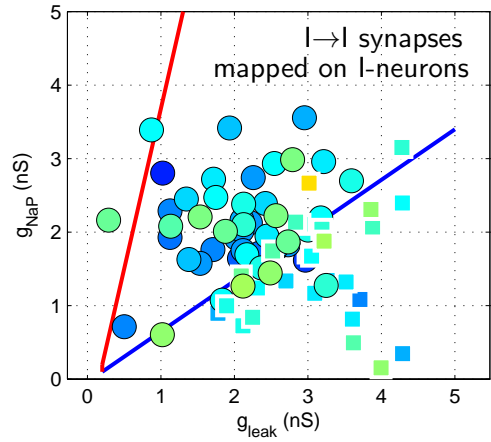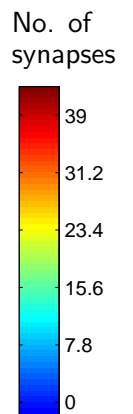

Supplement: S13 Fig — Synaptic distribution among neurons of evolved network for a different evolutionary run but with identical SynStrength and SynFrac values as in Fig 13. S14 Fig depicts the evolutionary history of this composite network. Initial synaptic distribution among neurons is randomly distributed with overall SynFrac = 0.06, which is lower than that of evolved network (SynFrac ≈ 0.10) and hence is not shown. (PDF) [file pone.0154049.s013.pdf]

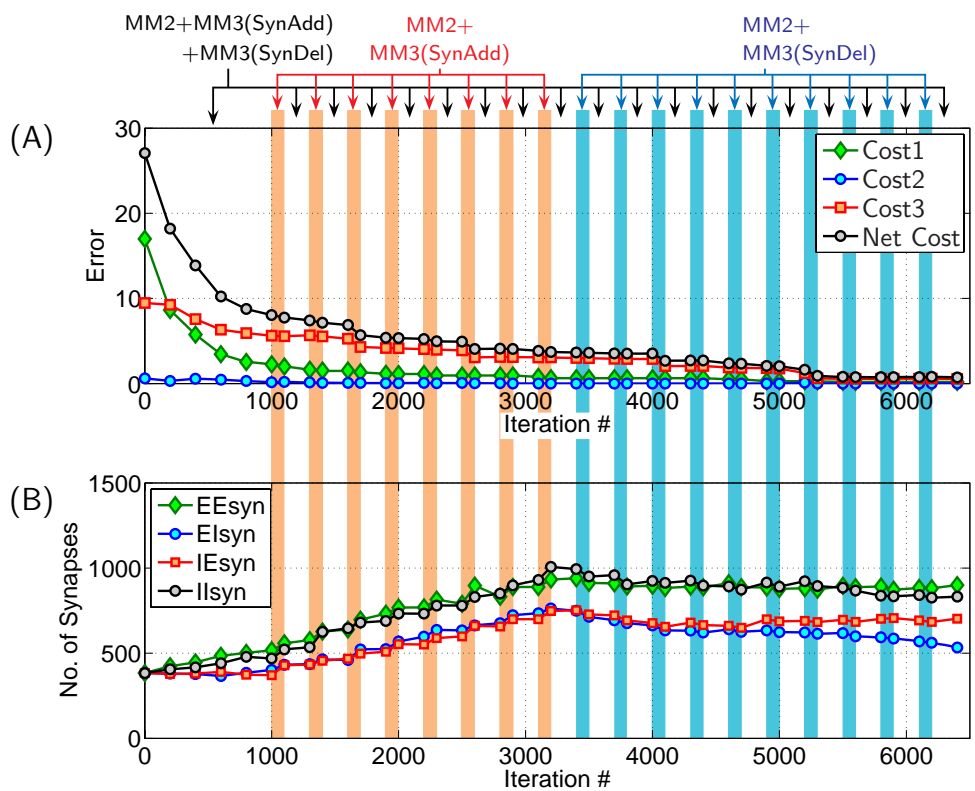

Supplement: S14 Fig — (A) Depiction of evolution of network in terms of ‘Cost’ minimization. Cost1, Cost2 and Cost3 are given by Eqs (1), (2) and (3), respectively. For network evolution, objective is to minimize (Cost1+Cost2+Cost3). Vertical color stripes indicates mutation methods that were employed during various stages for evolving the network. For further explanation see Appendix C. (B) Variation of synapses within the network during evolution process. Nomenclature: EEsyn, EIsyn, IEsyn and IIsyn are total synapses within EE, EI, IE and II part of adjacency matrix, see Fig 3C. (PDF) [file pone.0154049.s014.pdf]

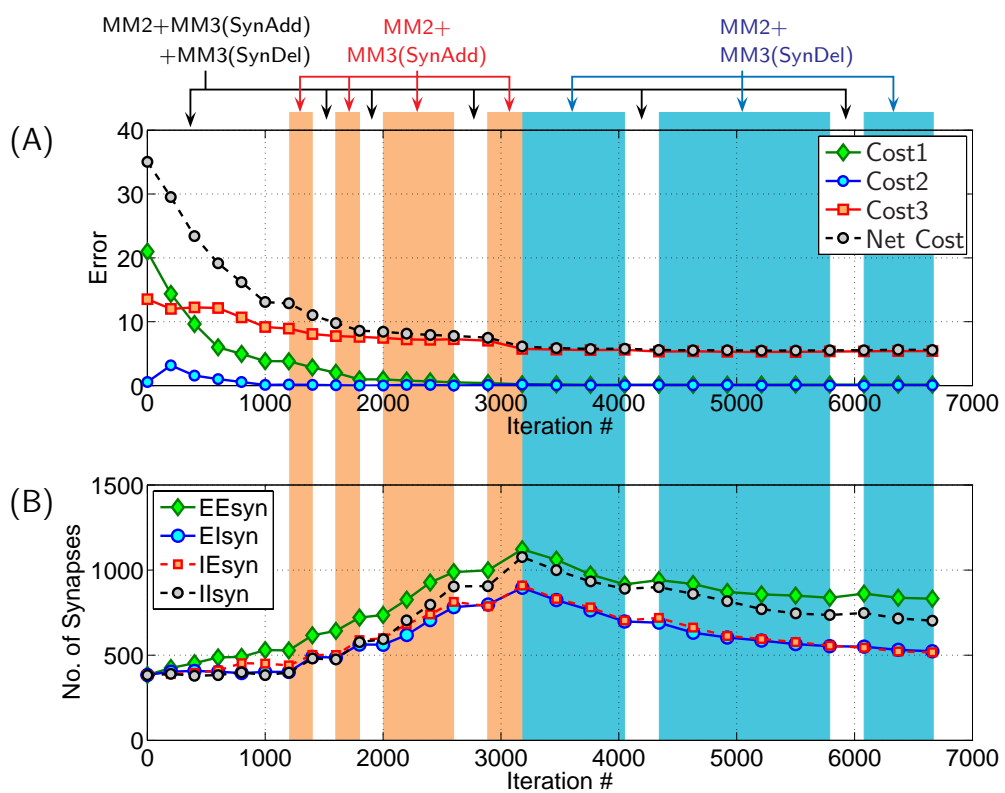

Supplement: S15 Fig — (A) Depiction of evolution of network in terms of ‘Cost’ minimization. Cost1, Cost2 and Cost3 are given by Eqs (1), (2) and (3), respectively. For network evolution, objective is to minimize (Cost1+Cost2+Cost3). Vertical color stripes indicates mutation methods that were employed during various stages for evolving the network. For further explanation see Appendix C. (B) Variation of synapses within the network during evolution process. Nomenclature: EEsyn, EIsyn, IEsyn and IIsyn are total synapses within EE, EI, IE and II part of adjacency matrix, see Fig 3C. (PDF) [file pone.0154049.s015.pdf]
