# Supplementary material for: Emergent Network Topology within the Respiratory Rhythm-Generating Kernel Evolved In Silico
Source: PLoS One. 2016 May 6;11(5):e0154049. doi: 10.1371/journal.pone.0154049 (PMC4859517; doi:10.1371/journal.pone.0154049)
Supplement: S9 Fig — Illustration for computing ‘Total sporadic burst count’ and ‘Total sporadic burst count possible’ required for computing Cost3 using Eq (3). ‘Left’: Raster plot for seven hypothetical neurons exhibiting different kind of bursting activities (remarked in rightmost column of table on the ‘Right’) in the interval between two successive population bursts. Time bins marked with grey colored raster data are counted as sporadic bursts. ‘Right’: Table indicating ‘total sporadic burst possible’ and ‘total sporadic burst actually exhibited’ by each neurons between the inter-burst interval. Refer Appendix B for further explanation. (PDF) [file pone.0154049.s009.pdf]

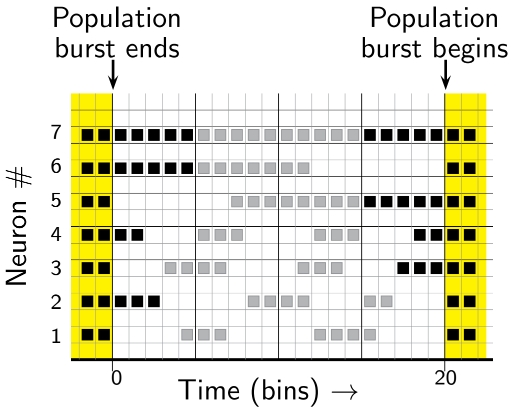

Neuron #	Total sporadic burst (bin) count possible	Total sporadic burst (bin) count	Remarks
7	10	10	Tonic neuron
6	15	7	neuron with elongated post-I activity
5	15	8	neuron with elongated pre-I activity
4	16	6	neuron with both short pre-I and short post-I activity
3	17	7	neuron with both short pre-I activity
2	17	6	neuron with both short post-I activity
1	20	7	
