# Supplementary material for: Emergent Network Topology within the Respiratory Rhythm-Generating Kernel Evolved In Silico
Source: PLoS One. 2016 May 6;11(5):e0154049. doi: 10.1371/journal.pone.0154049 (PMC4859517; doi:10.1371/journal.pone.0154049)
Supplement: S1 Table — (PDF) [file pone.0154049.s016.pdf]

	Type	Details	Remarks
**A**	Random Excitatory networks	- constituting neurons: 80 *excitatory* neurons with properties as depicted in Fig S5A,<br>- $SynFrac = 0.10$<br>- $SynStrength = 0.48$nS	Initialized random excitatory network used for evolving in section 2.5.
**B**	Random Excitatory networks	- constituting neurons: 80 *excitatory* neurons with properties as depicted in Fig S5B,<br>- $SynFrac = 0.06$<br>- $SynStrength = 0.60$nS	Excitatory sub-network of initialized random composite network used for evolving in section 2.6.
**C**	Random Excitatory networks	- constituting neurons: 80 *excitatory* neurons with properties as depicted in Fig S5B,<br>- $SynFrac = 0.12$<br>- $SynStrength = 0.60$nS	Excitatory sub-network of a random composite network with $SynFrac$ equal to that in *evolved* composite network (section 3.2).
**D**	Random Composite networks	- constituting neurons: 80 excitatory and 80 inhibitory neurons, properties of both 80 excitatory and 80 inhibitory neurons are as depicted in Fig S5B,<br>- $SynFrac = 0.06$<br>- $SynStrength = 0.60$nS	Initialized random composite network used for evolving in section 2.6.
**E**	Random Composite networks	- constituting neurons: 80 excitatory and 80 inhibitory neurons, properties of both 80 excitatory and 80 inhibitory neurons are as depicted in Fig S5B,<br>- $SynFrac = 0.06$<br>- $SynStrength = 0.60$nS	Random composite network with $SynFrac$ equal to that in *evolved* composite network (section 3.2).
