# Supplementary material for: Emergent Network Topology within the Respiratory Rhythm-Generating Kernel Evolved In Silico
Source: PLoS One. 2016 May 6;11(5):e0154049. doi: 10.1371/journal.pone.0154049 (PMC4859517; doi:10.1371/journal.pone.0154049)
Supplement: S1 Text — (PDF) [file pone.0154049.s017.pdf]

S1 Text

Intrinsic bursting frequency of constituting neurons and Network IBI

Intrinsic interburst periods of the 80 neurons depicted in Fig 5AC is depicted in S5 Fig. Note that intrinsic interburst period is the bursting period exhibited when neurons are synaptically isolated from each other. When these neurons are synaptically inter-connected to form a (neuronal) network, the network as a whole exhibits bursting. However, the interburst period of the network depends on the synaptic connection topology among the neurons. Thus, for different random synaptic connections (for a particular $SynFrac$ value) among the neurons, the network exhibits bursting with interburst period.

Histograms depicting distribution of inter-burst periods among random networks of different kinds are shown in S6 Fig; network details are provided in S1 Table.

It is worth pointing out that though the random networks A-E exhibit population bursts, the bursts are poorly synchronized (in general); typical population activity of networks A, D and F are depicted in Fig 6(Initial), Fig 12(Initial) and Fig 12(Random Network), respectively.
