# Supplementary material for: Emergent Network Topology within the Respiratory Rhythm-Generating Kernel Evolved In Silico
Source: PLoS One. 2016 May 6;11(5):e0154049. doi: 10.1371/journal.pone.0154049 (PMC4859517; doi:10.1371/journal.pone.0154049)
Supplement: S2 Text — (PDF) [file pone.0154049.s018.pdf]

S2 Text

Testing the effectiveness of MM4

Is it that the best evolved SW-network towards optimal synchronization (section 3.1.2, Fig 10) is limited by the mutation method algorithm MM4? To check this, the effectiveness of MM4 is tested for a *known* case. The methodology that we employed for this testing is as follows:

1. We took the connectivity matrix, say ConMat0, corresponding to the evolved excitatory network whose population activity is depicted as "Further evolved using MM3" in Fig 6, and reshuffled its elements by application of 50 consecutive mutations using MM4. Note that the connectivity matrix so obtained, say ConMat1, remains homeomorphic to ConMat0.

2. Keeping the neuronal properties of the constituting neurons unchanged and using ConMat1, we constructed an excitatory neuronal network, say NET1. Network activity of NET1 is depicted in S10 Fig 'Initial Net1'. Note that it is poorly synchronized.

3. Following this, network activity of NET1 was evolved to exhibit improved synchronization using random-hill climbing algorithm with MM4 as mutation method [note: the optimization methodology is exactly identical to method used to evolve the SW network in section 3.1.2 (see Fig. 10)]. Results are shown in S10 Fig. The improved synchronization exhibited by 'evolved NET1' is readily evident both in terms of neuronal activities (S10 Fig-ABC) and corresponding CC curves (S10 Fig-D).

4. Noting how the network connectivity of original (non-evolved) NET1 was constructed in step 1 above, it is clear that had the random-hill climbing algorithm using MM4 worked optimally then CC_{MM4} in S10 Fig-D should have matched CC_{MM3}. The gap between CC_{MM4} and CC_{MM3} in S10 Fig-D suggests sub-optimal optimization. However, the gap between CC_{MM4} and CC_{MM3} is small; this suggests that the random-hill climbing algorithm using MM4 for evolving network while preserving connectivity homeomorphism is reasonably effective.

In particular, the comparative large gap between CC_{MM4} and CC_{MM3} in Fig 10D, in comparison to that observed in S10 Fig-D, suggests that it is indeed *safe to conclude* that even the best evolved SW network provides suboptimal synchronization of neuronal network (in comparison to evolved excitatory network by method described in section 2.5).
