# Supplementary material for: Emergent Network Topology within the Respiratory Rhythm-Generating Kernel Evolved In Silico
Source: PLoS One. 2016 May 6;11(5):e0154049. doi: 10.1371/journal.pone.0154049 (PMC4859517; doi:10.1371/journal.pone.0154049)
Supplement: S3 Text — (PDF) [file pone.0154049.s019.pdf]

S3 Text: Manual explorations at evolving composite network employing Simulated Annealing Algorithm

S15 Fig depicts the details of various evolutionary stages of a typical network carried out manually; by 'manually' it is implied that the selection between MM3(SynAdd) and MM3(SynDel) during for evolving the network at various stages of its evolution is done manually. The evolutionary stages of the network may be divided into three different phases:

Iterations 0-1200: Initial stages of network evolution are carried out by employing generic simulation annealing algorithm, in which all three mutation methods – MM2, MM3(SynAdd) and MM3(SynDel) – are used for mutating the network. As the network continues to evolve, the Error (Error1+Error2+Error3) continues to diminish and reaches a stage where rate of decrease of Error per iteration becomes relatively small (iterations 1000-1200 in S15 Fig-A). This initial phase of network evolution is accompanied with increase in synapse count in the network (Fig 11B). Specifically, the synapse connecting excitatory to excitatory neurons increases relatively rapidly during this stage.

Iterations 1200-3180: To accelerate the rate of decrease of Error per iteration, and noticing the fact that the initial decrease in Error was accompanied with increase in network synapses, MM3(synDel) is intermittently deactivated in the simulated annealing algorithm. During the stages when MM3(synDel) is intermittently deactivated, the simulated annealing algorithm evolves the network using only MM2 and MM3(SynAdd). Notice the increased rate of decrease of Error during iterations 1200-1400 in comparison to between iterations 1000-1200.

In order to ensure that only Error-minimizing synapses are added to the network during the evolution process, MM3(synDel) is activated intermittently so that redundant synapses present in the network get discarded. (Note that, as explained in section 2.3, in case when two mutated networks perform equally well, i.e. result in same Error (Error1+Error2+Error3), the simulated annealing algorithm preferentially selects the network with lower synapse count.)

Moreover, MM2 remains always activated during the entire simulated annealing process, which ensure that if there is a mutation in which network's performance is improved by simple rewiring of exiting synapses (without any increase in synapse count), then such mutated networks are preferred.

After initial accelerated rate of decrease in Error, this process saturates – the rate of decrease of Error drops to almost zero. Addition of synapses to the network which doesn't result in decrease in Error are essentially redundant. Thus, we stop this process and move to next stage.

Iterations 3180-6660: Here the aim is to clear up the network of redundant synapses. So MM3(synAdd) is intermittently deactivated in the simulated annealing algorithm. During the stages when MM3(synAdd) is intermittently deactivated, the simulated annealing algorithm evolves the network using only MM2 and MM3(SynDel). Again, as explained in section 2.3, in case when two mutated networks perform equally well, the simulated annealing algorithm preferentially selects the network with lower synapse count. Thus, intermittent deactivation of MM3(synAdd) results in clearing up the network of redundant synapses.

Note that the rate of decrease in network's synapse count when MM3(synAdd) is intermittently deactivated not as high as rate of increase in network's synapse count when

MM3(synDel) is intermittently deactivated (iterations 1200-3180). Consequently, the intermittent ‘relaxing’ of network by employing all three mutation methods – MM2, MM3(SynAdd) and MM3(SynDel) – in simulated annealing is carried out less frequently during iterations 3180-6660 as compared to that during iterations 1200-3180.

Note that the presented evolutionary process of neuronal network comprises of only one cycle of increase and decrease in network's synapse count. Conventional paradigm of simulated annealing algorithm suggests execution of more than one such cycle. For the case depicted, we stopped after implementing one cycle for the following reasons:

1. The two iterations interval – 4050-4340 and 5790-6080, S15 Fig – during which the network was allowed to evolve in conjunction with MM3(SynAdd), network's synapse count increased without any attributable decrease in Error. This implies that the added synapses add no additional information regarding the nature of network connectivity pattern that is under investigation, and hence are redundant. Thus, any attempt to implement the next cycle of simulated annealing by first increasing and then decreasing network's synapse count to evolve the network further seems fruitless; for it requires addition of significant number of redundant synapse to the network first.

2. Care has been exercised to intermittently ‘relax’ the network during its evolution process so that the effect of random drift on evolutionary drift is minimal. For example, intermittently the network was cleared up of redundant synapses during increasing network's synapse count. Moreover, since MM2 remains always activated during the entire evolutionary process, it is ensured that if network's performance is improved by simple rewiring of exiting synapses (without any increase in synapse count), then such mutated networks are preferred.

3. Practical limitation of simulation time required: Total simulation time for data presented in S15 Fig is $\approx$(7.2min)$\times$(6660iterations). Repeating another cycle of evolutionary process would require almost equal amount of additional time.
